# Dbl2 Regulates Rad51 and DNA Joint Molecule Metabolism to Ensure Proper Meiotic Chromosome Segregation

**DOI:** 10.1371/journal.pgen.1006102

**Published:** 2016-06-15

**Authors:** Silvia Polakova, Lucia Molnarova, Randy W. Hyppa, Zsigmond Benko, Ivana Misova, Alexander Schleiffer, Gerald R. Smith, Juraj Gregan

**Affiliations:** 1 Department of Membrane Biochemistry, Institute of Animal Biochemistry and Genetics, Slovak Academy of Sciences, Ivanka pri Dunaji, Slovakia; 2 Department of Chromosome Biology, Max F. Perutz Laboratories (MFPL), University of Vienna, Vienna, Austria; 3 Department of Genetics, Faculty of Natural Sciences, Comenius University, Bratislava, Slovakia; 4 Division of Basic Sciences, Fred Hutchinson Cancer Research Center, Seattle, Washington, United States of America; 5 IMP/IMBA Bioinformatics Core Facility, Research Institute of Molecular Pathology (IMP), Vienna Biocenter, Vienna, Austria; National Cancer Institute, UNITED STATES

## Abstract

To identify new proteins required for faithful meiotic chromosome segregation, we screened a *Schizosaccharomyces pombe* deletion mutant library and found that deletion of the *dbl2* gene led to missegregation of chromosomes during meiosis. Analyses of both live and fixed cells showed that *dbl2Δ* mutant cells frequently failed to segregate homologous chromosomes to opposite poles during meiosis I. Removing Rec12 (Spo11 homolog) to eliminate meiotic DNA double-strand breaks (DSBs) suppressed the segregation defect in *dbl2Δ* cells, indicating that Dbl2 acts after the initiation of meiotic recombination. Analyses of DSBs and Holliday junctions revealed no significant defect in their formation or processing in *dbl2Δ* mutant cells, although some Rec12-dependent DNA joint molecules persisted late in meiosis. Failure to segregate chromosomes in the absence of Dbl2 correlated with persistent Rad51 foci, and deletion of *rad51* or genes encoding Rad51 mediators also suppressed the segregation defect of *dbl2Δ*. Formation of foci of Fbh1, an F-box helicase that efficiently dismantles Rad51-DNA filaments, was impaired in *dbl2Δ* cells. Our results suggest that Dbl2 is a novel regulator of Fbh1 and thereby Rad51-dependent DSB repair required for proper meiotic chromosome segregation and viable sex cell formation. The wide conservation of these proteins suggests that our results apply to many species.

## Author Summary

Meiosis produces haploid gametes from diploid precursor cells. This reduction of chromosome number is achieved by two successive divisions after only a single round of DNA replication. To identify novel regulators of meiosis, we screened a library of fission yeast deletion mutants and found that deletion of the *dbl2* gene led to missegregation of chromosomes during meiosis. Analysis of live *dbl2Δ* cells by fluorescence microscopy showed that chromosomes frequently failed to segregate during the first meiotic division. Further cytological and biochemical analyses revealed that this segregation defect is due to persistent intermediates of DNA double-strand break repair, also called DNA joint molecules. Our results indicate that Dbl2 is required for formation of Fbh1 DNA helicase foci at the sites of DNA double-strand break repair in order to process DNA joint molecules and allow segregation of chromosomes during meiotic divisions. Our bioinformatics searches revealed that Dbl2 is highly conserved in fungi, animals and plants, suggesting that Dbl2 plays a similar role in other organisms–the formation of viable sex cells and healthy progeny.

## Introduction

During meiosis, haploid gametes are produced from diploid precursor cells. The reduction of chromosome number is achieved by a single round of DNA replication followed by two rounds of chromosome segregation, termed meiosis I and meiosis II. While meiosis II is similar to mitosis in that sister centromeres segregate from each other, centromeres of homologous chromosomes (homologs) segregate to opposite poles in meiosis I [1,2].

Three meiosis-specific features are essential for proper segregation of chromosomes during meiosis I–formation of crossovers that connect homologs, mono-orientation of sister kinetochores, and a stepwise loss of sister chromatid cohesion. The formation of crossovers, as a result of meiotic recombination, and the attachment of sister kinetochores to microtubules emanating from the same spindle pole (mono-orientation) ensure that homologous centromeres are pulled in opposite directions on meiosis I spindles [2,3].

Crossovers and cohesion between sister chromatids distal to crossovers are responsible for holding homologs together until the onset of anaphase I, when a protease called separase cleaves cohesin along chromosome arms [4–6]. This allows segregation of recombined homologs to opposite poles of the meiosis I spindle. During meiosis I, cleavage of centromeric cohesin is blocked by Sgo1 (called Mei-S332 in *Drosophila*) complexed with the protein phosphatase 2A (PP2A) [7–9]. Deprotection of centromeric cohesin and a second round of separase activation allow cleavage of the centromeric cohesin at the onset of anaphase II, which is followed by segregation of sister centromeres in meiosis II [10].

Homologous recombination involves programmed formation of DNA double-strand breaks (DSBs) and an evolutionarily conserved pathway for DSB repair, which operates in both mitotic and meiotic cells. To promote high-level recombination (including crossovers) in meiosis, programmed DSBs are made by the highly conserved topoisomerase-like protein Spo11 (called Rec12 in the fission yeast *Schizosaccharomyces pombe*) and several essential accessory factors [11–13]. These meiotic DSBs are repaired using the intact sister chromatid or the homolog as a template [14]. Only recombination between homologs can lead to formation of the crossovers required for proper segregation of chromosomes during meiosis I. During DSB formation, Rec12 becomes covalently attached to DNA 5’ ends and is subsequently removed by an endonuclease, Mre11-Rad50-Nbs1 (MRN) complexed with Ctp1 [15–17]. The DNA 5′ ends are further resected to generate long 3′ single-stranded DNA (ssDNA) overhangs [18]. These ssDNA ends are then coated by Rad51 and (in some species) meiosis-specific Dmc1, both of which are homologs of the bacterial DNA strand-exchange protein RecA [19]. Rad51 promotes the formation of DNA joint molecules (JMs) between Rad51-ssDNA filaments and homologous double-stranded (ds) DNA [20]. Auxiliary proteins, called “recombination mediators”, such as Rad52, Rad54, Rad55, Rad57, Sfr1, Swi5, and Rdh54, promote the formation and/or stabilization of Rad51-ssDNA filaments [20,21]. Other proteins, such as the F-box DNA helicase Fbh1 and (in the budding yeast *Saccharomyces cerevisiae*) the Srs2 helicase, are *negative* regulators of Rad51. Members of the Swi2/Snf2 family of DNA motor proteins, Rad54 and Rdh54, enhance Rad51-mediated formation of JMs but are also involved in the removal of Rad51 from DNA, suggesting that JM metabolism needs to be carefully regulated [22–26]. Rad51-ssDNA filaments invade homologous dsDNA to form a displacement loop (D-loop). Subsequent DNA synthesis primed by the invading 3’ DNA end extends the invading strand [20]. The recombination reaction can then take one of two different paths. If the extended invading strand is displaced and anneals with the other DSB end, a non-crossover is produced in a hypothetical process called synthesis-dependent strand-annealing (SDSA). Alternatively, the strand invasion intermediate is stabilized, and capture of the second DSB end leads to formation of a Holliday junction (HJ) [14]. HJs can be resolved by endonucleolytic activities such as Mus81-Eme1, which is critical in *S*. *pombe*, where it is the only known complex involved in meiotic HJ resolution [27–29].

Regulation of the formation and processing of meiotic JMs, the subject of this report, is complex and incompletely understood. We report here a new role for Dbl2, which was first identified in a screen for *S*. *pombe* mutants defective in chromosome segregation during meiosis [30]. It was later identified in a screen for proteins forming microscopic foci at HO endonuclease-induced DSBs [31]. Dbl2 is required for normal DSB targeting of the DNA-repair helicase Fml1 [31]. Here, we show that Dbl2 is required for proper segregation of chromosomes during meiosis by regulating Rad51 function and JM metabolism, apparently by promoting formation of the helicase Fbh1 foci at the sites of DSB repair to dissociate a minor class of JMs. We discuss molecular mechanisms by which JM formation and processing are properly regulated for successful meiosis and the conservation of these proteins among species.

## Results

### Dbl2 is required for proper segregation of chromosomes during meiosis and mitosis

**Fig 1:**
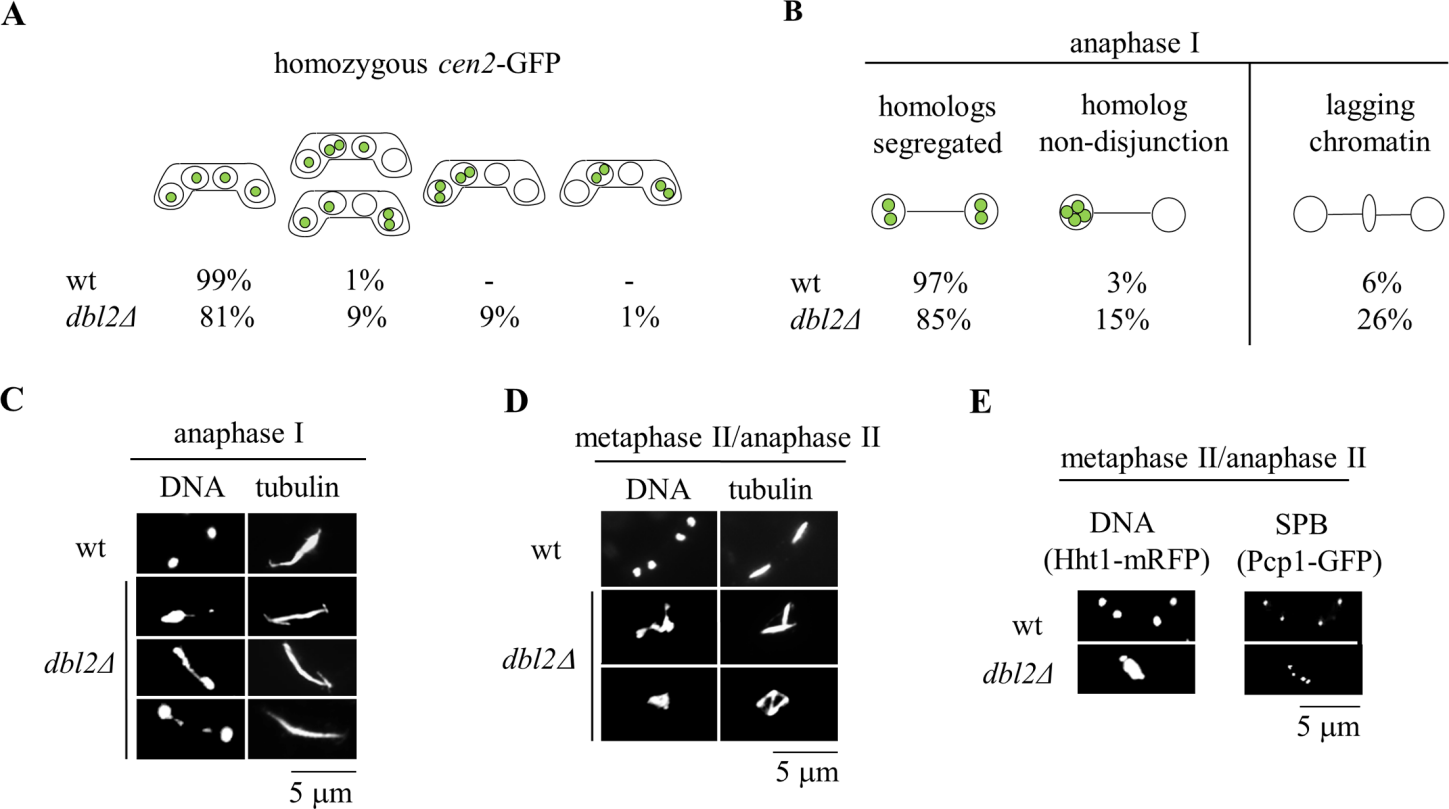
Dbl2 is required for proper segregation of chromosomes during meiosis I. **(A)** The segregation of chromosome 2 was scored in wild-type (JG12618) and *dbl2Δ* (JG17130) *h*^*90*^ strains in which chromosome 2 was marked with GFP (*cen2*-GFP). The strains were induced for meiosis and at 20–24 hr fixed and DNA was visualized by Hoechst staining. Chromosome 2 segregation (*cen2*-GFP dots) was scored in 500 asci. **(B)** The strains described in (A) were immunostained for tubulin and GFP; DNA was visualized by DAPI. *cen2-*GFP dots and lagging chromatin were scored under the fluorescence microscope in 100 anaphase I cells. **(C)** Examples of anaphase I zygotes in wild-type and *dbl2Δ* mutant, showing lagging chromatin and unsegregated DNA. **(D)** The images show spindle and chromatin morphology at metaphase II or anaphase II in wild type (JG15456 x JG11318) and *dbl2Δ* mutant (JG17208 x JG17207) meiosis. Cells were fixed and immunostained for tubulin; DNA was visualized by Hoechst staining. Mononucleate *dbl2Δ* zygotes with two or three spindles are shown. **(E)** Spindle pole bodies (SPBs) and chromosomes were visualized by endogenously tagged Pcp1-GFP and Hht1-mRFP, respectively. In a wild-type strain (JG16917), four SPBs are present at the completion of meiosis II, and each is associated with one of the four nuclei. In the *dbl2Δ* mutant (JG17116), mononucleate zygotes containing up to four SPBs are observed.

To identify novel proteins required for faithful meiotic chromosome segregation, we screened a library of about 3200 *S*. *pombe* deletion mutants purchased from Bioneer. We found that deletion of the *dbl2* gene frequently led to missegregation of chromosomes during meiosis. The *dbl2* gene was also identified in our previous screening in which we deleted 180 functionally uncharacterized genes whose expression is upregulated during meiosis and screened for mutants defective in meiotic chromosome segregation [30]. To confirm that this phenotype is due to deletion of *dbl2*, we deleted the *dbl2* gene in a haploid homothallic (*h*^*90*^) strain in which the centromere of chromosome 2 was marked with GFP (*cen2*-GFP [32]). We sporulated mutant cells, stained nuclei with DAPI and scored segregation of GFP dots in asci with four nuclei. Indeed, we found that *dbl2Δ* mutant cells frequently missegregated chromosomes during meiosis (Fig 1A).

To investigate chromosome segregation directly in anaphase cells, we fixed cells and stained with antibodies against tubulin and GFP. In wild-type cells, homologous centromeres segregate to opposite poles during anaphase I. However, we frequently observed non-disjunction of homologous centromeres and lagging chromosomes in *dbl2Δ* anaphase I cells (Fig 1B and 1C). In the majority of anaphase I cells with lagging chromosomes, telomeres of chromosome 1 (*sod2*-GFP signals) lagged, while centromeres of chromosome 2 (*cen2*-GFP signals) segregated to the poles (S1 Fig). This indicates that in *dbl2Δ* mutant cells microtubules are frequently able to attach to kinetochores and pull the sister kinetochores to opposite poles, while the chromosomal arms and telomeres lag behind. Although we have analyzed only telomeres of chromosome 1 and centromeres of chromosome 2, we suppose that this is representative also for other chromosomes and thus it is unlikely that chromosomes in *dbl2Δ* mutant cells lag because they do not attach to microtubules.

**Fig 2:**
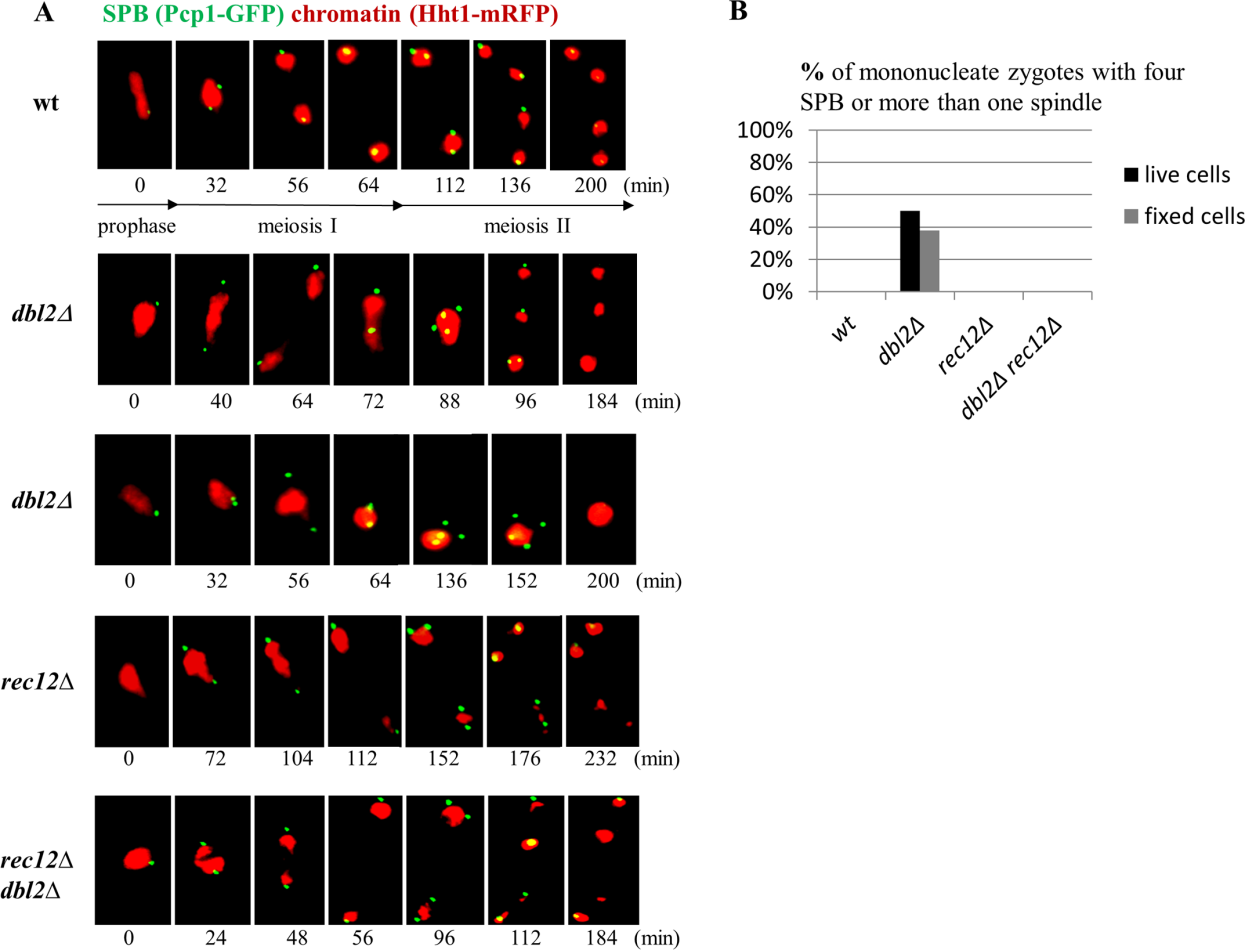
Deletion of *dbl2* causes a Rec12-dependent failure of chromosome segregation during meiosis I. **(A)** A wild-type strain (JG16917), *dbl2Δ* mutant (JG17116), *rec12Δ* mutant (JG17159) and *rec12Δ dbl2Δ* double mutant (JG17156) were plated on SPA sporulation plates and analyzed by live-cell imaging. The SPBs and chromosomes were observed via endogenously tagged Pcp1-GFP and Hht1-mRFP, respectively. Numbers below the images represent time, in minutes, elapsed since filming began at the end of the horsetail stage. Prophase, meiosis I and meiosis II are indicated. **(B)** The graph shows the percentage of live cells with four SPBs or more than one spindle on a single DNA mass (examples of such zygotes are shown in panel A) and the percentage of fixed cells with two or more spindles on a single DNA mass (examples of such zygotes are shown in Fig 1D). In live-cell imaging, 20 wild-type, 26 *dbl2Δ*, 10 *rec12Δ* and 17 *dbl2Δ rec12Δ* zygotes were scored. For the analysis of fixed cells, the strains (JG12618, JG17130, JG17351, JG17353) were immunostained for tubulin, DNA was visualized by Hoechst staining, and 100 zygotes from each strain were scored.

In addition, in *dbl2Δ* mutant cells we frequently observed mononucleate cells containing one spindle and stretched but undivided chromatin (Fig 1C) as well as mononucleate cells containing more than one spindle or four spindle pole bodies (SPBs) (Fig 1D and 1E). This result suggests that despite the failure to segregate chromosomes at meiosis I, *dbl2Δ* cells proceeded to form metaphase II spindles within a single nucleus. Live-cell imaging of *dbl2Δ* cells confirmed chromosome missegregation and failure to segregate chromosomes to opposite poles during meiosis I (Fig 2A). Remarkably, deletion of *rec12* (*spo11* homolog) suppressed the meiosis I chromosome segregation failure in *dbl2Δ* cells (Fig 2). In 13 out of 26 *dbl2Δ* zygotes observed, chromosomes failed to segregate to opposite poles during meiosis I, whereas in all 17 *dbl2Δ rec12Δ* zygotes observed, chromosomes segregated during meiosis I (Fig 2A and 2B). The suppression of the failure of meiosis I chromosome segregation in *dbl2Δ rec12Δ* zygotes was observed in both live and fixed cells (Fig 2B). Because Rec12 is required for meiotic DSB formation and recombination [11,33,34], these results imply that Dbl2 acts after the initiation of meiotic recombination to allow faithful chromosome disjunction.

Failure to segregate chromosomes in meiosis I could also be caused by an inability to remove cohesin from chromosome arms, which physically links two homologs that have recombined until the onset of anaphase I [6,35,36]. However, we found no evidence for defective removal of cohesin in *dbl2Δ* mutant cells when we analyzed the Rec8-GFP cohesin subunit, which is cleaved to allow cohesin removal from chromosomes [35] (S2 Fig).

**Fig 3:**
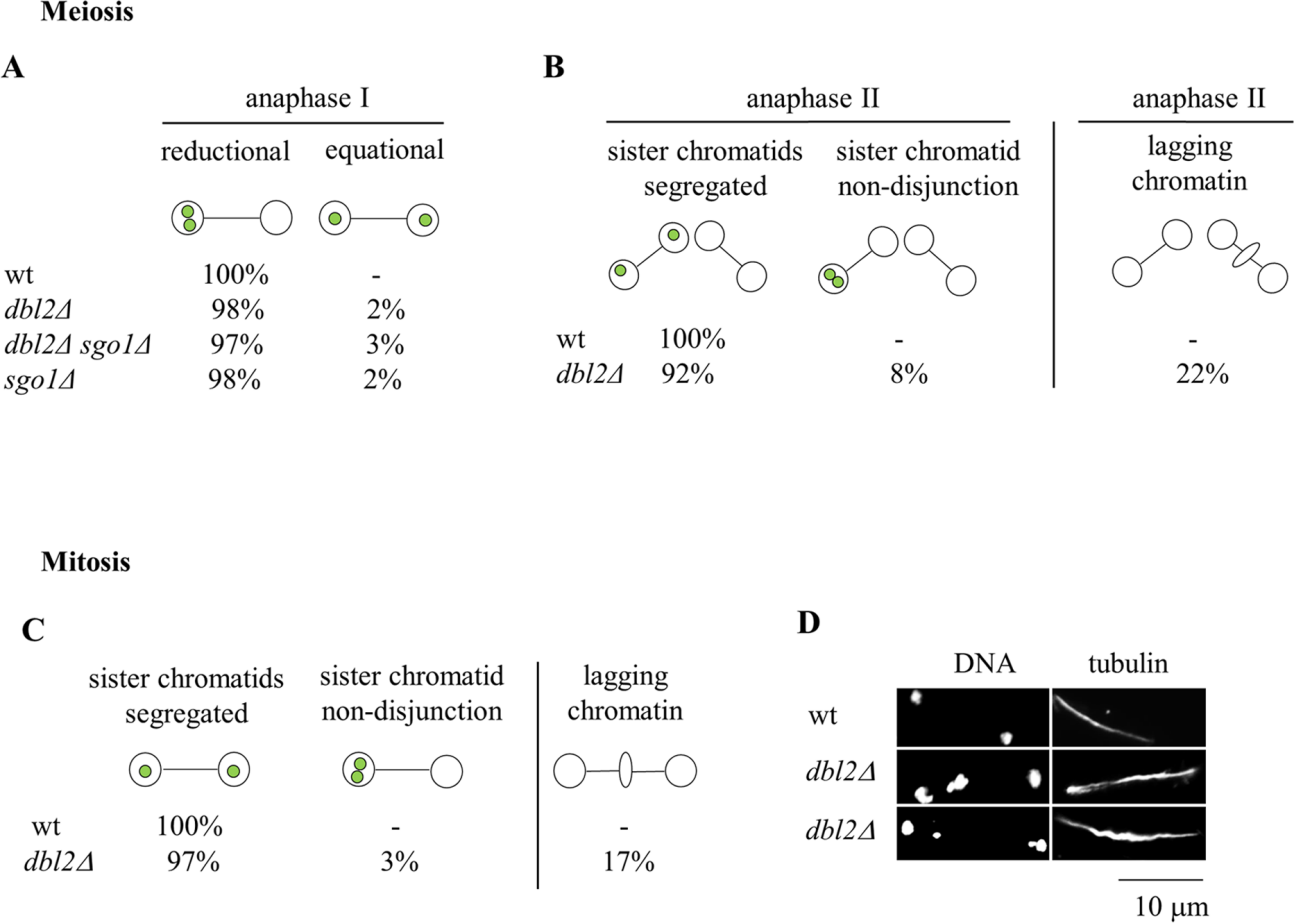
Dbl2 is required for proper segregation of sister chromatids during meiosis II and mitosis. **(A)** *h*^*+*^ strains carrying *cen2*-GFP that were either wild-type (JG15456), *dbl2Δ* (JG17208), *sgo1Δ* (JG12269) or *dbl2Δ sgo1Δ* (JG17779) were crossed to *h*^*-*^ strains of the same genotype but lacking *cen2*-GFP (JG11318, JG17207, JG11793 and JG17780). Cells were fixed and immunostained for tubulin and GFP; DNA was visualized by Hoechst staining. The segregation of chromosome 2 was scored in 100 anaphase I cells. **(B)** *h*^*+*^ *cen2*-GFP strains either wild-type (JG15456) or *dbl2Δ* (JG17208), were crossed to *h*^*-*^ strains of the same genotype but lacking *cen2*-GFP (JG11318, JG17207). Zygotes were fixed and processed as in (A). The segregation of chromosome 2 and lagging chromatin were scored in 100 anaphase II cells. **(C)** The segregation of chromosome 2 was scored in mitotically dividing wild-type (JG15456) and *dbl2Δ* mutant (JG17208) cells. Cells were fixed and processed as in (A). The segregation of chromosome 2 and lagging chromatin were scored in 100 anaphase cells. **(D)** Examples of a wild-type anaphase cell and two *dbl2Δ* mutant cells showing lagging chromatin during anaphase.

Failure of chromosome segregation in meiosis I has also been observed in mutants defective in mono-orientation of sister kinetochores. In this case, centromeric sister chromatid cohesion, which persists throughout the first meiotic division, prevents segregation of bi-oriented sister chromatids to opposite poles [37–41]. Mutants defective in mono-orientation attempt but fail to divide nuclei during the first meiotic division; elimination of centromeric cohesion allows them to undergo an equational meiosis I division [37–41]. If meiosis I nuclear division failure in *dbl2Δ* mutant cells were due to a defect in mono-orientation of sister kinetochores, then elimination of centromeric cohesion at the onset of anaphase I should allow *dbl2Δ* mutant cells to undergo an equational meiosis I division. Elimination of the centromeric cohesin protector Sgo1 in a *dbl2Δ* strain with only one copy of chromosome 2 marked with GFP (*cen2*-GFP) did not increase equational segregation of sister centromeres during meiosis I (Fig 3A). Moreover, we frequently observed mononucleate cells containing more than one spindle in *dbl2Δ sgo1Δ* mutant cells, indicating that the meiosis I nuclear division failure was not suppressed by elimination of Sgo1. These results are consistent with the idea that in *dbl2Δ* mutant cells the failure to segregate chromosomes during meiosis I is not caused by a defect in mono-orientation of sister kinetochores.

In addition to defects in meiosis I, we observed a high frequency of lagging chromosomes and missegregation of sister centromeres in *dbl2Δ* cells during anaphase II and mitosis (Fig 3B–3D). Thus, in addition to segregation of homologs during meiosis I, Dbl2 is required for proper segregation of sister chromatids during both meiosis II and mitosis.

### Meiotic DSBs and Holliday junctions are formed and processed normally in *dbl2Δ* mutant cells

Our observation that the meiosis I chromosome segregation failure in *dbl2Δ* is suppressed by *rec12Δ* as well as a previous report that Dbl2 binds to DSBs to facilitate targeting of DNA repair helicase Fml1 [31] prompted us to analyze the role of Dbl2 in the formation of asci and viable spores and in recombination.

**Fig 4:**
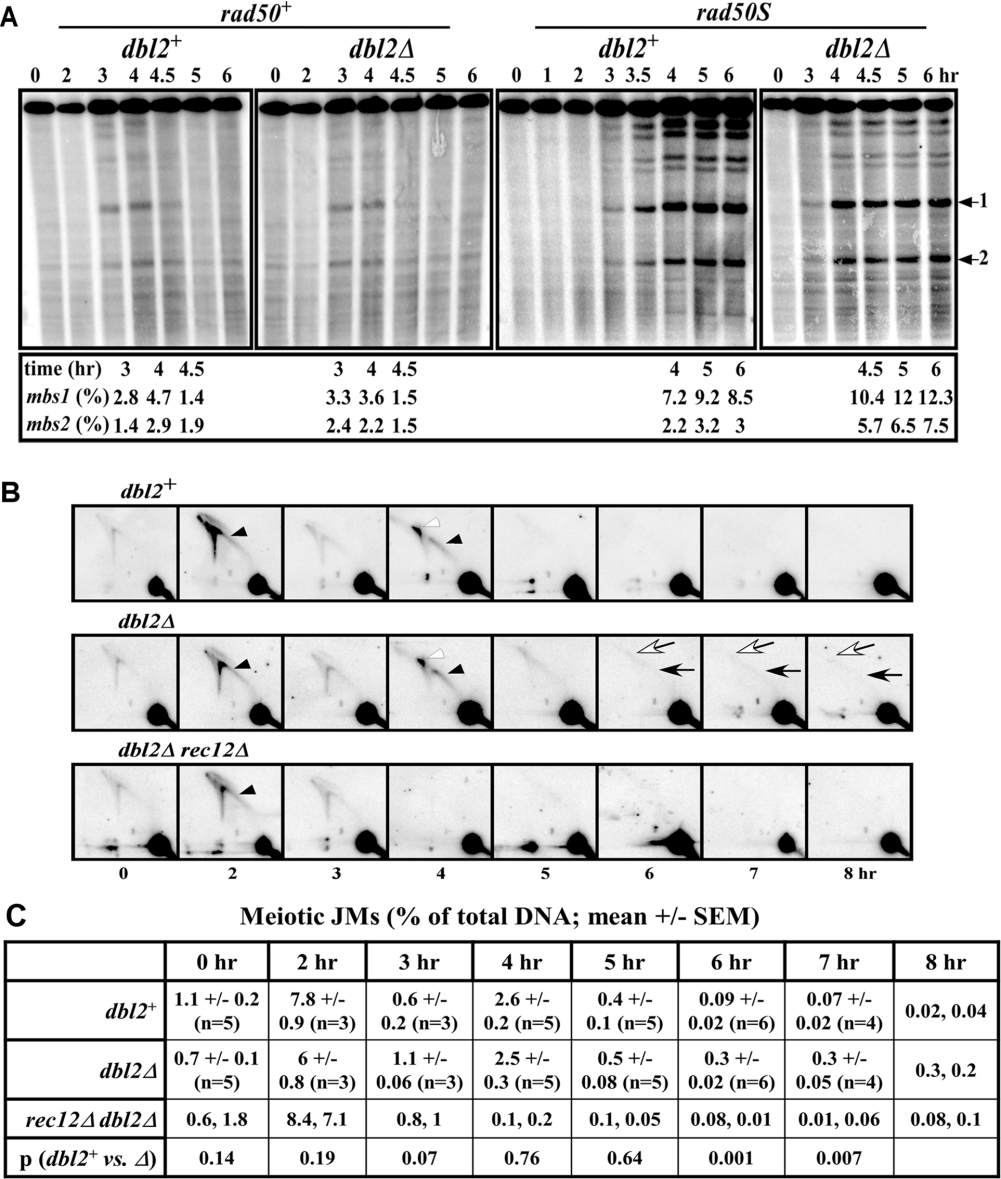
DNA double-strand breaks and Holliday junctions are formed and repaired similarly in wild-type and *dbl2Δ* mutant, but Rec12-dependent joint molecules persist in *dbl2Δ* late meiosis. **(A)** Formation and repair of DSBs on the 0.5 Mb *Not*I fragment J. Strains GP6656 (*dbl2*^*+*^), GP8664 (*dbl2Δ*) and GP8450 (*dbl2Δ rad50S*) were induced for meiosis; DNA was prepared at the indicated times, digested with *Not*I, and analyzed by pulsed-field gel electrophoresis and Southern blot hybridization using a probe at the left end of the 501 kb *Not*I fragment J (band at the top of the gel) [42]. GP3718 (*dbl2*^*+*^ *rad50S*) is from [94]. The fraction of total DNA broken at *mbs1* and at *mbs2* (indicated by arrows on the right) at the indicated time for each strain is shown below each blot. The timing of appearance and disappearance of DSBs and their frequencies in both *rad50*^*+*^ and *rad50S* strains are similar to those previously reported in wild-type cells [42,95]. **(B)** Formation and resolution of DNA joint molecules at the *mbs1* hotspot. Strains GP6656 (*dbl2*^*+*^), GP8664 (*dbl2Δ*), and GP8836 (*dbl2Δ rec12Δ*) were induced for meiosis; DNA was extracted at the indicated times, digested with *Bsr*GI, and analyzed by two-dimensional gel electrophoresis and Southern blot hybridization using a probe (~1 kb long) near the *mbs1* DSB hotspot on the 10.5 kb *Bsr*GI fragment (major spot at the bottom right) [96]. Black and white arrowheads, respectively, indicate Y-arc and X-spike species (replication and recombination intermediates) found transiently in wild-type cells. Black and white arrows indicate Y-arc and X-spike species, respectively, that persist only in *dbl2Δ rec12*^*+*^ cells (at 6, 7, and 8 hr). **(C)** Quantification of branched DNA (JMs) in panel B. ImageQuant analysis was used to quantify the amount of branched DNA (Y-arc plus X-spike) relative to the total DNA. Data are the mean ± SEM from n assays (*dbl2*^*+*^ and *dbl2Δ*) or individual data from duplicate assays (*rec12Δ dbl2Δ*). See S2 Table for values of all individual data. p values, from unpaired t-tests, are the probability that *dbl2*^*+*^ and *dbl2Δ* do not differ at the indicated time points. See S4 Fig and S3 Table for additional data at *mbs1* and at the *ade6-3049* DSB hotspot on a different chromosome.

The *dbl2Δ* mutant produced abnormal asci, which often contained fewer than four spores or unequal-size spores (S3 Fig), in accord with the frequent chromosome missegregation noted above. The overall yield of viable spores per cell in the mating mixture was about 13 times less in the *dbl2Δ* mutant than in wild type (Table 1), indicating a severe defect in meiosis. Viability of the few spores produced was about 57% of that of wild type (Table 2). However, the frequency of both intergenic (*ade6* –*arg1*) and intragenic (*ade6*) recombination in viable spores was similar to that in wild-type (Table 1). As expected from the nearly wild-type levels of recombination, Southern blot hybridizations of DNA extracted from *dbl2Δ* mutant cells induced for meiosis revealed no defect in the formation and disappearance of DSBs at six DSB hotspots spanning a 0.50 Mb interval, *mbs1* and *mbs2* being the most prominent (Figs 4A and S4). In *rad50*^*+*^ strains the timing of appearance and disappearance of DSBs was similar to that in wild type; in *rad50S* strains, in which DSB repair is blocked, DSBs accumulated to similar levels in wild type and the *dbl2* mutant [27,42]. Similarly, Holliday junction intermediates of meiotic recombination were formed and resolved with nearly wild-type kinetics (Figs 4B, 4C and S4). These results are consistent with the nearly wild-type recombination levels in the *dbl2* mutant.

**Table 1:** Reduced yield of viable spores but normal recombination in the absence of Dbl2.

	*dbl2*^*+*^	*dbl2∆*
Viable spore yield^a^	8.5 ± 0.95	0.64 ± 0.055
Ade^+^/million viable spores	2150 ± 95	2875 ± 300
*ade6 –arg1* recombinants	45% (61/136)	36% (48/132)

Strains crossed were GP13 and GP1293 (*dbl2*^*+*^), and GP8696 and GP8698 (*dbl2*::*natMX4*). Two independent cultures of each parent were mated in all four combinations. Spore titrations produced nearly equal numbers of light red (*ade6-52*) and dark red (*ade6-M26*) colonies. Reciprocal intergenic recombinant frequencies were nearly equal. Data were homogeneous and were pooled. Data are mean ± SEM; n = 4. For intragenic recombination, p = 0.058 by t test. For intergenic recombination, p = 0.098 by Fisher’s exact test. Viable spore yield per input cell of the minority parent is greater than two because of slight growth of cells before mating and meiosis.

^a^ Viable spores/viable input cell of the minority parent.

**Table 2:** Genetic interaction between *dbl2Δ* and mutations in homologous recombination-related genes.

Strain	Zygotes with Rad51 foci		Mononucleate zygotes with more than one spindle (%)	Spore viability (%)
	Anaphase I (%)	Anaphase II (%)		
wt	< 1	< 1	< 1	95 ± 1.5
*dbl2Δ*	62.6 ± 1.5	63 ± 0.9	47 ± 1.2	57 ± 6.4
*fbh1Δ*	88.3 ± 1.2	86 ± 2	92 ± 3.2	20 ± 4.1
*fbh1Δ dbl2Δ*	91 ± 2.3	87.6 ± 1.4	93 ± 0.9	16 ± 3.5
*rad51Δ*	< 1	< 1	< 1	< 1
*rad51Δ dbl2Δ*	< 1	< 1	< 1	< 1
*rad52Δ*	< 1	< 1	< 1	36 ± 4.9
*rad52Δ dbl2Δ*	6.6 ± 0.9	< 1	< 1	26 ± 6.4
*rad57Δ*	< 1	< 1	< 1	25 ± 7
*rad57Δ dbl2Δ*	8 ± 1.7	3 ± 1.2	< 1	39 ± 14.5
*rad55Δ*	< 1	< 1	< 1	31 ± 6
*rad55Δ dbl2Δ*	10 ± 1.5	< 1	< 1	59 ± 8.4
*sfr1Δ*	< 1	< 1	< 1	79 ± 3.7
*sfr1Δ dbl2Δ*	7.6 ± 1.7	< 1	< 1	82 ± 2.7
*rad54Δ*	10 ± 1.2	< 1	< 1	32 ± 7.2
*rad54Δ dbl2Δ*	12 ± 2.3	< 1	< 1	33 ± 9.7
*dmc1Δ*	< 1	n.d.	< 1	n.d.
*dmc1Δ dbl2Δ*	59 ± 2.9	n.d.	42 ± 1.7	n.d.
*rdh54Δ*	< 1	n.d.	< 1	n.d.
*rdh54Δ dbl2Δ*	63 ± 1.7	n.d.	44 ± 0.9	n.d.
*fml1Δ*	< 1	n.d.	< 1	n.d.
*fml1Δ dbl2Δ*	61 ± 0.9	n.d.	45 ± 1.7	n.d.
*fml2Δ*	< 1	n.d.	< 1	n.d.
*fml2Δ dbl2Δ*	61 ± 1.2	n.d.	40 ± 1.4	n.d.

Single mutants were *fbh1Δ* (JG17544), *dbl2Δ* (JG17146), *rad51Δ* (JG17540 x JG17506), *rad57Δ* (JG17749 x JG17750), *rad55Δ* (JG17756 x JG17755), *rad52Δ* (JG17823 x JG17824), *sfr1Δ* (JG17746), *rad54Δ* (JG17815 x JG17817), *dmc1Δ* (JG17513), *rdh54Δ* (JG17716), *fml1Δ* (JG17784), *fml2 Δ* (JG17788) and a wild-type (JG11355). Double mutants were *fbh1Δ dbl2Δ*, (JG17545), *rad51Δ dbl2Δ* (JG17542 x JG17507), *rad57Δ dbl2Δ* (JG17751 x JG17752), *rad55Δ dbl2Δ* (JG17757 x JG17758), *rad52Δ dbl2Δ* (JG17747 x JG17748), *sfr1Δ dbl2Δ* (JG17811), *rad54Δ dbl2Δ* (JG17821 x JG17819), *dmc1Δ* (JG17511), *rdh54Δ* (JG17718), *fml1Δ* (JG17786), *fml2Δ* (JG17790). Data are means ± SEM of three independent experiments. Cells were fixed at 10–17 hr after meiotic induction and immunostained for tubulin and Rad51; DNA was visualized by Hoechst staining. At least 100 cells and 500 spores were scored. We cannot exclude the possibility that spore viability in *fbh1Δ* strains was affected by suppressor mutations which may occur in *fbh1Δ* mutant cells [23].

n.d., not determined

We noted that a low level of joint molecules (JMs) persisted in the *dbl2* mutant longer than in wild type (Fig 4B and 4C). JMs persisted for at least 8 hr after meiotic induction in *dbl2Δ* but were scarcely detectable at or after 6 hr in *dbl2*^*+*^. Accumulation of these species was Rec12-dependent, indicating that these JMs arise from meiotic DSBs and might be related to recombination intermediates. Since many of these JMs migrated on the arc that includes Y-shaped molecules, such as replication forks, we suspect they are D-loops, or closely related structures, which are postulated to be precursors to Holliday junctions (see Discussion) [27]. These persistent JMs could account for the failure of chromosome segregation and lagging chromosomes noted above (Figs 1–3). These persistent JMs were seen at two different DSB hotspots: *mbs1* (Fig 4B) and *ade6-3049* (S4 Fig).

**Fig 5:**
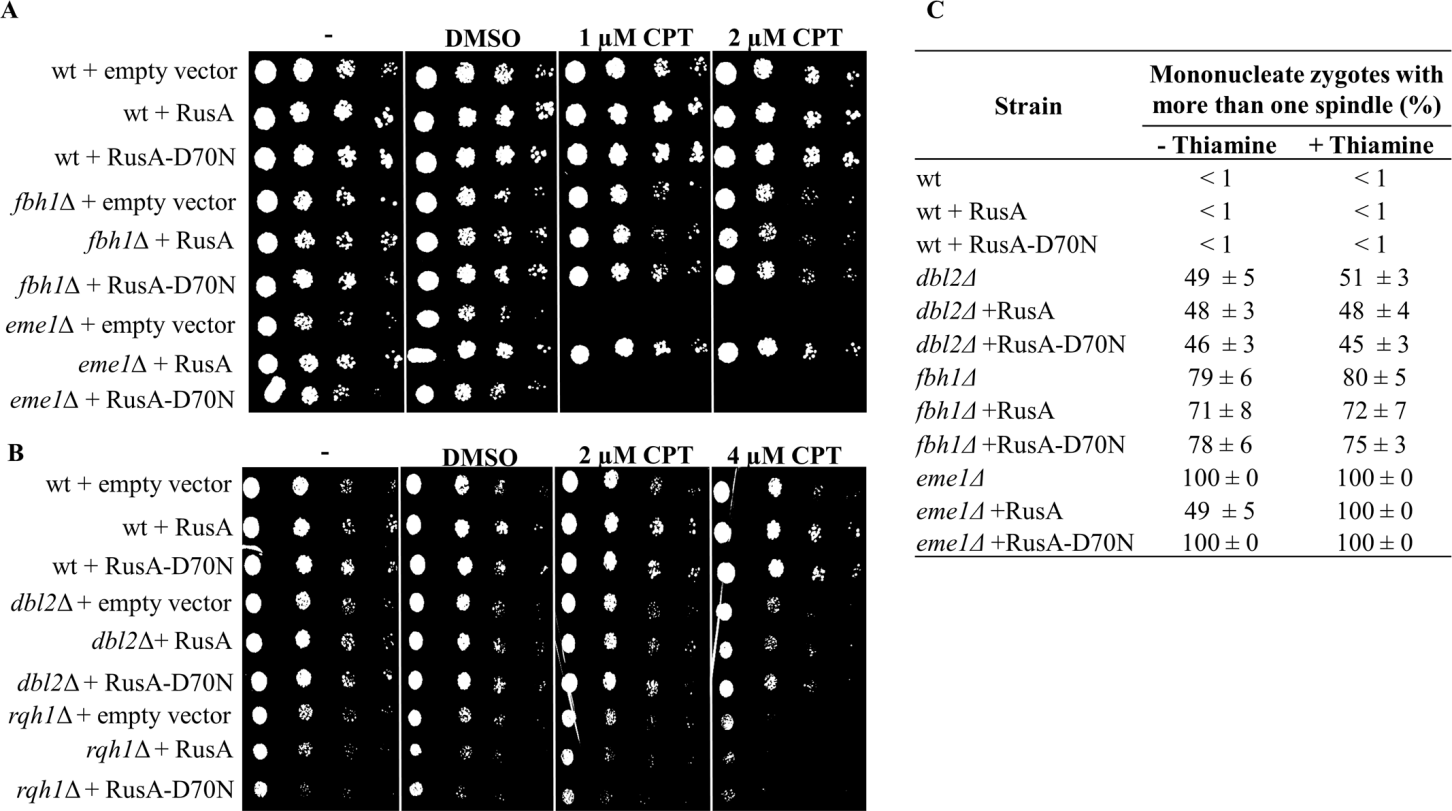
Expression of *E*. *coli* Holliday junction resolvase RusA does not suppress the camptothecin sensitivity or chromosome segregation defect of *dbl2Δ* mutant cells. **(A** and **B)** Wild-type, *dbl2*∆, *eme1∆*, *fbh1∆* and *rqh1∆* strains were transformed with either pREP41 plasmid (empty vector), resulting in strains JG17953, JG17955, JG17959, JG17957 and JG17944, or pREP41-RusA plasmid, resulting in strains JG17453, JG17451, JG17444, JG17945 and JG17448, or pREP41-RusA-D70N plasmid, resulting in strains JG17452, JG17450, JG17445, JG17947 and JG17449. Cells were grown on EMM2 liquid medium without leucine for one day, diluted in 5-fold steps, and spotted onto EMM2 plates lacking thiamine and containing the indicated amounts of camptothecin (CPT). RusA and RusA-D70N were expressed under the control of a thiamine-repressible *nmt1*-promotor using a pREP41 vector [97]. pREP41-RusA-D70N, expressing an inactive version (D70N) of RusA [98], and an empty vector were used as negative controls. Plates were incubated at 32°C for 4 days and photographed. **(C)** Strains described in panels A and B and strains JG11355, JG17146, JG17544, JG17827, JG17949 and JG17951 were grown with or without thiamine (15 μM), fixed and immunostained for tubulin and GFP. DNA was visualized by Hoechst staining. 100 zygotes with more than one spindle on a single DNA mass were scored in three independent experiments. Data are means ± SEM of three independent experiments.

Although defects in processing Holliday junctions can lead to chromosome segregation defects [27,28,43,44], we think it is unlikely that this is the case in *dbl2Δ* mutant cells. Consistent with the idea that Dbl2 has a function independent of the Mus81-Eme1 Holliday junction resolvase [27,28,43,44] is the synthetic mitotic growth defect of a *dbl2Δ eme1Δ* double mutant strain: the double mutant grew much more poorly than either single mutant (S5 Fig). Moreover, expression of a wild-type *E*. *coli* Holliday junction resolvase RusA but not the nuclease-dead RusA-D70N mutant suppressed camptothecin-sensitivity of *eme1Δ* mutant cells, as previously reported, but no suppression was seen in *fbh1Δ*, *rqh1Δ* (lacking the Rqh1 DNA repair helicase [45]) or *dbl2Δ* mutant cells (Fig 5A and 5B) [28,43]. RusA expression failed to suppress the meiosis I chromosome segregation defect in *dbl2Δ* and *fbh1Δ* mutant zygotes but partially suppressed the *eme1Δ* mutant phenotype as assessed by scoring mononucleate zygotes containing more than one spindle (Fig 5C). These data indicate that Dbl2 does not have a major role in DSB formation or disappearance, Holliday junction formation or resolution, or the formation of recombinants. This prompted us to investigate other roles of Dbl2 in meiotic recombination.

### Dbl2 is required for timely removal of Rad51 nucleofilaments

**Fig 6:**
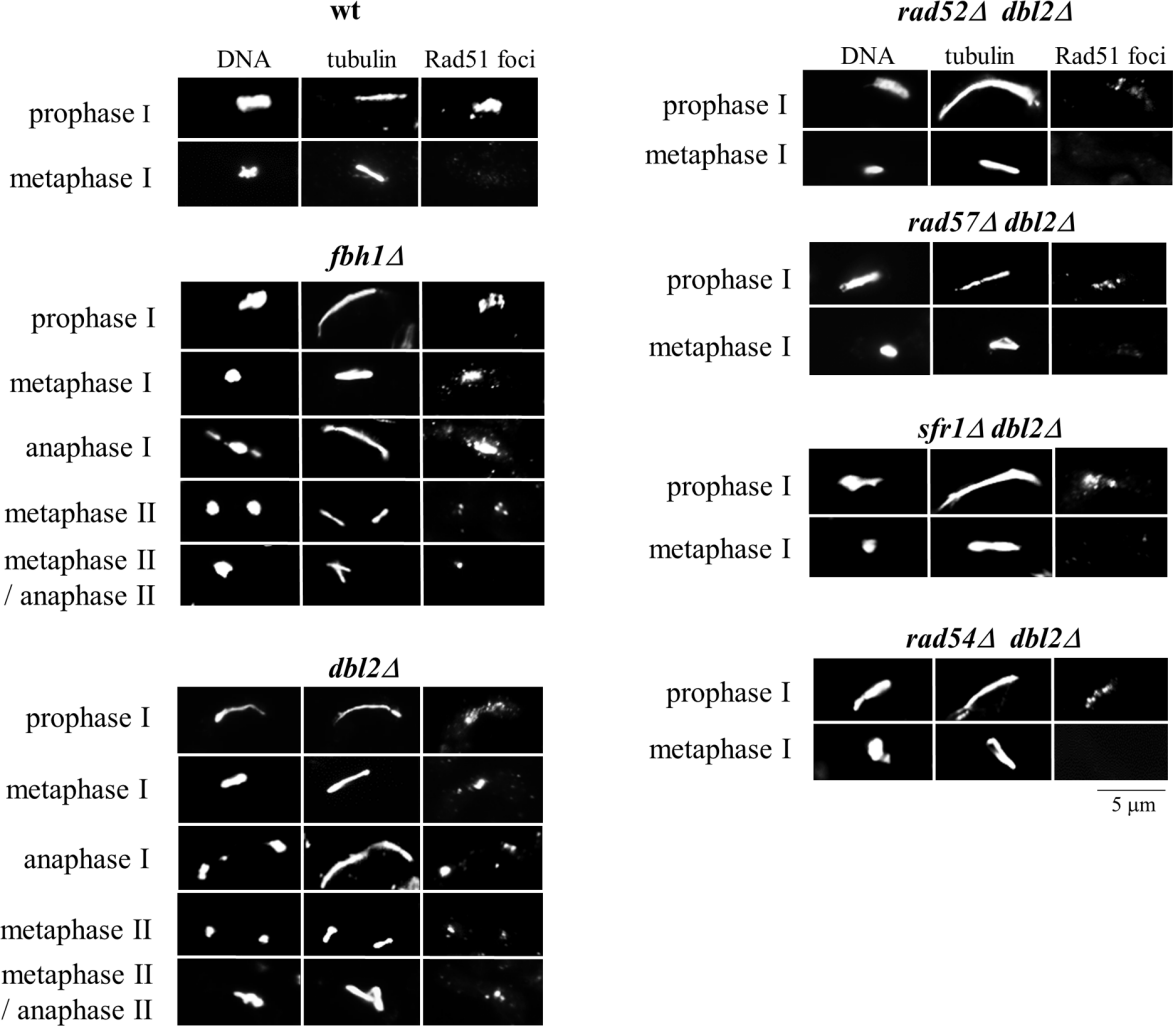
Failure to segregate chromosomes in the absence of Dbl2 correlates with persistent Rad51 foci. Cells were mated on SPA sporulation agar and at 10–17 hr fixed and immunostained for tubulin and Rad51; DNA was visualized by Hoechst staining. Representative images show that Rad51 foci persisted until anaphase II in *fbh1Δ* (JG17544) and *dbl2Δ* (JG17146) mutant cells but not in wild type (JG11355). Deletion of *rad52*, *rad57*, *sfr1* or *rad54* restored nearly wild-type frequencies of nuclei with levels of Rad51 foci in the *dbl2Δ* mutant cells. The strains were *rad57Δ dbl2Δ* (JG17751 x JG17752), *rad52Δ dbl2Δ* (JG17747 x JG17748), *sfr1Δ dbl2Δ* (JG17811), *rad54Δ dbl2Δ* (JG17821 x JG17819). No Rad51-GFP foci were detected in anaphase I or anaphase II zygotes of single mutants *rad57Δ* (JG17749 x JG17750), *rad52Δ* (JG17823 x JG17824), *sfr1Δ* (JG17746), *rad54Δ* (JG17815 x JG17817) and wild-type (JG11355) (see Table 2).

Homologous recombination is promoted by Rad51, which forms filaments with ssDNA that undergo strand exchange with homologous dsDNA molecules [19,20]. The formation and timely disassembly of Rad51-ssDNA filaments is essential for successful DNA repair by homologous recombination. In wild-type *S*. *pombe* cells, Rad51 foci are present during meiotic prophase, when DSBs are formed and repaired, but are no longer present in metaphase I cells [46]. In the absence of proteins required for timely removal of Rad51-ssDNA filaments, such as the F-box DNA helicase Fbh1, Rad51 foci persist throughout both meiotic divisions [23]. We used an antibody raised against fission yeast Rad51 to visualize formation of Rad51 foci in meiotic cells. Interestingly, in *dbl2Δ* mutant cells Rad51 foci formed during meiotic prophase and, as in *fbh1Δ* mutant cells, remained visible during meiosis I and meiosis II and in mononucleate cells containing more than one spindle (Fig 6, Table 2). Thus, the failure to segregate chromosomes in the absence of Dbl2 correlates with persistent Rad51 foci.

### Deletion of *rad51* suppresses the chromosome segregation defect in *dbl2Δ* mutant cells

If the failure to segregate chromosomes in the *dbl2Δ* mutant were due to a defect in removal of Rad51-ssDNA filaments or Rad51-dependent JMs, then elimination of Rad51 should allow *dbl2Δ* mutant cells to segregate chromosomes during meiosis I, though perhaps improperly due to unrepaired DSBs or reduced crossover numbers. Indeed, no mononucleate zygotes containing more than one spindle were observed in a *dbl2Δ rad51Δ* double mutant (Table 2). A similar suppression of the *dbl2Δ* mutation was observed upon deletion of any one of the genes encoding mediator proteins Rad52, Rad55, Rad57, Sfr1, and Rad54 that promote Rad51-ssDNA filament formation [47,48]; such suppression was not observed upon deletion of *negative* regulators of Rad51 function (Fml1 or Fml2), the meiosis-specific Rad51 paralog Dmc1, or Rdh54 (Table 2). Consistent with the notion that the chromosome segregation defect in *dbl2Δ* mutant cells is Rad51-dependent, the synthetic lethality of the *rqh1Δ dbl2Δ* double mutant was suppressed by deletion of *rad51* in mitotic cells. These data suggest that Rad51 prevents proper segregation of chromosomes in the absence of Dbl2.

### Dbl2 is required for efficient formation of Fbh1 foci in CPT-induced DNA lesions

Our observation that Rad51 foci persist in *dbl2Δ* mutant cells beyond meiotic prophase raises the possibility that Dbl2 regulates proteins responsible for disassembly of Rad51-ssDNA filaments. Fbh1, an F-box helicase related to bacterial UvrD, negatively regulates Rad51-mediated homologous recombination by disrupting Rad51 nucleoprotein filaments [22]. Interestingly, the *fbh1Δ* mutant phenotype resembles that of *dbl2Δ–*meiotic DSB formation and repair as well as recombination are close to wild-type levels, chromosomes frequently fail to segregate during meiosis I, Rad51 foci persist beyond meiotic prophase and the *fbh1Δ* mutant phenotype is suppressed by *rad51Δ*, *rad52Δ* or *rad57Δ* [23,49–51].

**Fig 7:**
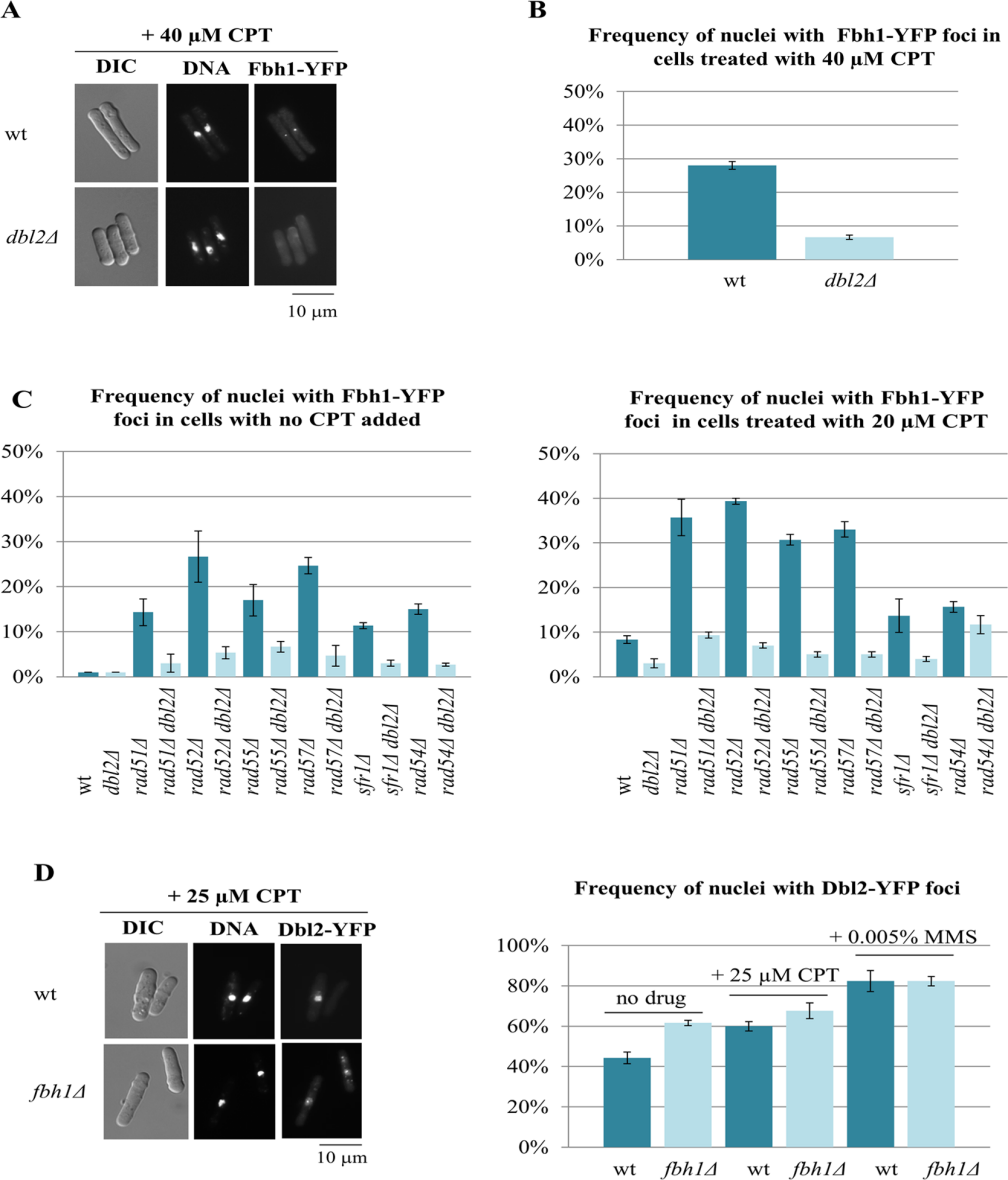
Dbl2 is required for efficient formation of Fbh1 foci at DNA lesions induced by CPT or deletion of genes involved in homologous recombination. **(A** and **B)** *S*. *pombe* strains expressing Fbh1-YFP from plasmid pMW651 and carrying *fbh1Δ* (JG17775) or *fbh1Δ dbl2Δ* (JG17777) mutations growing in EMM2 medium without leucine at 32°C were treated with CPT (40 μM) for 4 hr and fixed; DNA was visualized with DAPI. The *dbl2Δ* mutant showed significantly fewer number of Fbh1-YFP foci in G2 cells compared to those in *dbl2*^*+*^. The values reported are means of three independent experiments ± SEM. Fbh1-YFP foci were scored in 200 G2 cells. Values for each individual experiment are shown in S4 Table. **(C)** Strains were grown at 32°C for 4 hr in EMM2 medium without leucine and without (left panel) or with (right panel) 20 μM CPT and processed as in (A). The strains used were wild-type (JG17843), *dbl2Δ* (JG17844), *rad51Δ* (17837), *rad51Δ dbl2Δ* (JG17838), *rad52Δ* (JG17839), *rad52Δ dbl2Δ* (JG17840), *rad55Δ* (JG17833), *rad55Δ dbl2Δ* (JG17834), *rad57Δ* (JG17835), *rad57Δ dbl2Δ* (JG17836), *sfr1Δ* (JG17831), *sfr1Δ dbl2Δ* (JG17832), *rad54Δ* (JG17841), *rad54Δ dbl2Δ* (JG17842). The values reported are means of three independent experiments ± SEM. In each experiment 200 G2 cells were scored. Values for each individual experiment are shown in S5 Table. **(D)** *S*. *pombe* wild-type strain (JG17962) and *fbh1Δ* mutant (JG17961) expressing Dbl2-YFP were grown in EMM2 medium without leucine and treated with either 25 μM CPT or 0.005% MMS for 4 hr and fixed; DNA was visualized with DAPI. Dbl2-YFP foci were scored in three sets of 200 G2 cells. Values for each individual experiment are shown in S6 Table. See S6 and S7 Figs for additional data.

We therefore tested whether Fbh1 foci, which co-localize with Rad51 foci after meiotic DSB formation [23], are affected in *dbl2Δ* mutant cells. Because Fbh1 is not visible when tagged at the endogenous locus, we used a strain in which the endogenous *fbh1* gene was deleted and the yellow fluorescent protein (YFP)-tagged Fbh1 was expressed from a strong *nmt* promoter [23]. Fbh1-YFP forms foci in response to Rec12-dependent DSBs that co-localize with Rad51 foci, suggesting that this construct is functional [23]. We used camptothecin (CPT), a topoisomerase I inhibitor, to induce DNA lesions in vegetative cells [52]. Fbh1-YFP foci were visible in *dbl2*^*+*^ cells but were strongly reduced in *dbl2Δ* mutant cells (Fig 7A and 7B). This reduction was not due to a higher frequency of plasmid loss in *dbl2Δ* mutant cells because the stability of the Fbh1-YFP plasmid was similar in both *dbl2*^*+*^ cells (5.8% plasmid loss per generation) and *dbl2Δ* cells (6.7% plasmid loss per generation). We obtained similar results when we induced DNA lesions in vegetative cells using methyl methanesulfonate (MMS) [53,54] (S6 Fig). Fbh1-YFP foci were also visible in *rad51Δ*, *rad52Δ*, *rad55Δ*, *rad57Δ*, *sfr1Δ* and *rad54Δ* mutant strains, but the frequency of Fbh1-YFP foci was decreased by the *dbl2Δ* mutation in each of these strains, both in the absence and presence of CPT (Fig 7C). The reduction of Fbh1-YFP foci in *dbl2Δ* mutant cells was not due to reduced levels of DNA lesions because Rad52-mCherry foci, which represent sites of active DNA repair [55], were not reduced in *dbl2Δ* mutant cells (S7 Fig).

To investigate whether localization of Dbl2 and Fbh1 to DSBs are interdependent, we analyzed Dbl2-YFP expressed from a strong *nmt* promoter [56]. A previous report showed that Dbl2-YFP formed a focus at an HO endonuclease-induced DSB [31]. We observed that Dbl2-YFP formed nuclear foci in wild-type cells when DNA lesions were induced by MMS or CPT (Fig 7D, S6 Table). Deletion of *fbh1* had no effect on the formation of Dbl2 foci (Fig 7D, S6 Table). This suggests that Dbl2-YFP expressed from a strong *nmt* promoter is able to form foci in the absence of Fbh1. However, we cannot exclude the possibility that this is due to overexpression of Dbl2-YFP or the presence of spontaneous suppressor mutations which may occur in *fbh1Δ* mutant cells [23].

**Fig 8:**
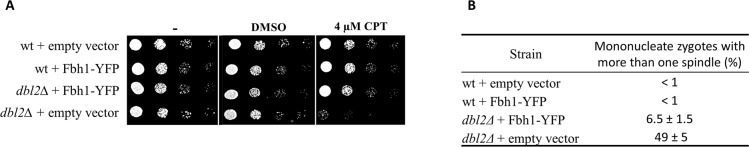
Overexpression of Fbh1 suppresses the *dbl2Δ* mutant phenotype. **(A)** Wild-type and *dbl2Δ* strains expressing either Fbh1-YFP (JG18021, JG18022) or empty vector (pREP41) (JG17953, JG17955) were grown in EMM2 liquid medium for one day, diluted in 5-fold steps, and spotted onto EMM2 plates lacking thiamine and containing the indicated amounts of camptothecin (CPT). The plates were photographed after 4 days of incubation at 32°C. **(B)** Strains described in panel (A) were mated on SPA sporulation plates, fixed after 10–17 hr and immunostained for tubulin. DNA was visualized by DAPI. Mononucleate zygotes with more than one spindle were scored in three sets of 100 meiotic cells. The values reported are means of three independent experiments ± SEM.

If the role of Dbl2 were to promote accumulation of Fbh1 at DNA lesions, we would expect overexpression of Fbh1 to suppress the *dbl2Δ* mutant phenotype. Indeed, both CPT-sensitivity and the meiotic chromosome segregation defect in *dbl2Δ* mutant cells were nearly fully suppressed by expression of Fbh1-YFP from a strong *nmt* promoter (Fig 8A and 8B).

These data suggest that Dbl2 promotes accumulation of Fbh1 at DNA lesions, such as DSBs, independently of recombination proteins Rad51, Rad52, Rad55, Rad57, Sfr1 and Rad54.

## Discussion

### A critical role for Dbl2 in chromosome segregation

Dbl2 was identified in screenings for mutants defective in chromosome segregation during meiosis [30] and for proteins that localize to DNA double-strand breaks (DSBs) [31]. Here, we report that in the absence of Dbl2, Rad51 foci and Rad51-dependent DSB-repair intermediates (DNA joint molecules, or JMs) persist and frequently prevent proper segregation of chromosomes during meiosis I. Also in the absence of Dbl2, foci of the F-box helicase Fbh1 are less abundant than in the presence of Dbl2. We propose that a subset of JMs requires Fbh1 for their reversal or processing into Holliday junctions (HJs) resolvable by Mus81-Eme1 and that Dbl2 is required to promote formation of Fbh1 foci at these JMs. As predicted, the phenotypes of *dbl2Δ* and *fbh1Δ* are similar, although not identical, as discussed below. These proteins, like the formation of JMs during meiosis, are widely conserved, suggesting that diverse species require Dbl2 for successful meiosis and reproduction.

### Dbl2 promotes formation of Fbh1 foci to process a rare class of DNA joint molecules

The data reported here can be accounted for by the following proposal. During DSB repair, a subset of JMs is not converted into HJs. Some of these JMs might be D-loops, since they migrate on the "Y-arc" characteristic of such JMs (Figs 4 and S4). Others might be related to HJs, such as hemicatenanes, since they migrate on the "X-arc" characteristic of such JMs. (Figs 4 and S4). The helicase Fbh1, recruited by Dbl2, either reverses these JMs to allow synthesis-dependent strand annealing (SDSA) or enables their extension and conversion into HJs, which can be resolved by the Mus81-Eme1 HJ resolvase. This proposal accounts for our observations as follows.

We observed a rare class of JMs that persisted late in meiosis in *dbl2Δ* cells (Figs 4B, 4C and S4). These JMs account for about 0.3% of the total DNA in a 12 kb interval containing the *mbs1* or the *ade6-3049* DSB hotspot (Figs 4C and S4), which corresponds to about 3 JMs per cell. This estimate is uncertain, because the low level of these JMs is not far above the background level and because the density (number per kb) of these JMs might be less in DSB-cold regions. Furthermore, some of the persistent JMs detected might not block segregation. Nevertheless, this frequency of persistent JMs is compatible with the frequency of missegregating or lagging chromosomes or cells with one nucleus but two spindles, which are seen in 20–50% of cells (Figs 1, 2 and 3; Table 2); if Poisson distributed, an average of one persistent JM per cell would result in 63% of cells with failed chromosome segregation. Thus, these persistent JMs are likely the cause of the failure of chromosomes to segregate properly during both meiosis I and II, consistent with *rec12Δ* eliminating both the persistent JMs (Figs 4 and S4) and chromosome segregation failure (Fig 2). These JMs might be between sisters or between homologs, but for the reasons below, we suspect they are primarily intersister JMs. During meiosis I, when homologous centromeres segregate, a persistent intersister JM distal to a crossover would prevent segregation in a similar way that non-cleaved cohesin mediating sister chromatid cohesion prevents segregation [35,57,58]. A persistent interhomolog JM would also prevent segregation. During meiosis II, when sister centromeres segregate, an intersister JM would directly prevent segregation if not aided by a DNA helicase, such as Fbh1.

Other meiotic phenotypes of *dbl2Δ* are accounted for by this proposal. The suppression, by elimination of DSBs (*rec12Δ*), of the *dbl2Δ* chromosome segregation failure (Fig 2) and of persistent JM formation (Figs 4B, 4C and S4) is accounted for by the requirement for Rec12 and DSBs to form meiotic JMs. Similarly, the suppression of both Rad51-focus accumulation and chromosome segregation failure in *dbl2Δ* mutants by *rad52Δ*, *rad55Δ*, *rad57Δ*, *sfr1Δ*, and *rad54Δ* is accounted for by the enhancement of Rad51 strand exchange by the corresponding proteins (Table 2). It is particularly noteworthy that *dmc1Δ* does not suppress *dbl2Δ* but *rad52Δ* does (Table 2). Dmc1 is not required for formation of intersister JMs at the loci tested in *S*. *pombe* [59] or in *S*. *cerevisiae* [60], and genetic data indicate that *S*. *pombe* Rad52 is required for intersister but not interhomolog JM formation [61]. Furthermore, purified *S*. *pombe* Rad52 stimulates Rad51 strand exchange, although it inhibits Dmc1 strand exchange [62,63]. Failure to convert intersister JMs into HJs would not reduce recombinant frequencies but would prevent proper chromosome segregation and decrease viable spore yield, as observed in *dbl2Δ* (Table 1). Failure to recruit Fbh1 to JMs in *dbl2Δ* cells would allow accumulation of Rad51 foci (Fig 6; Table 2) and rare JMs (Figs 4B and S4).

The mitotic phenotypes of *dbl2Δ* are similarly accounted for. After DNA damage, the appearance of Fbh1 foci is Dbl2-dependent (Fig 7). *dbl2Δ* mutants are sensitive to camptothecin (CPT) (Fig 5), which inhibits topoisomerase 1 and leaves DNA lesions that must be repaired [52]. Mitotic DNA repair is thought to be primarily with the sister chromatid [64]. If some intersister JMs other than HJs, such as D-loops and hemicatenanes, persist in *dbl2Δ* mutants, expression of the bacterial RusA HJ resolvase would not alleviate the problem, as observed (Fig 5). In contrast, the CPT-sensitivity and presumed accumulation of HJs in *eme1Δ* mutants, which lack the *S*. *pombe* HJ resolvase Mus81-Eme1 [28], is suppressed by expression of catalytically active RusA (Fig 5).

The phenotypes of *dbl2Δ* and *fbh1Δ* are remarkably similar. In both mutants during meiosis, DSB formation and repair as well as recombination are close to wild-type levels, chromosomes frequently fail to segregate during meiosis I, Rad51 foci persist beyond meiotic prophase, and chromosome missegregation is suppressed by *rad51Δ*, *rad52Δ* or *rad57Δ* (Figs 1, 2, 3, 4, 6 and S1; Table 2) [23,51]. The slightly higher spore viability and milder chromosome segregation defects in *dbl2Δ* than in *fbh1Δ* may result from residual Dbl2-independent binding of Fbh1 to DSBs or JMs. Moreover, during mitotic growth, both *dbl2Δ* and *fbh1Δ* mutant cells are sensitive to camptothecin [65] and show negative genetic interaction (phenotype of double mutant is stronger than single mutants) with mutants defective in DNA repair such as *srs2Δ* and *rsc4Δ* and positive genetic interaction (suppression of the mutant phenotype) with *rad55Δ* and *rad57Δ* [66].

It is interesting that Dbl2 promotes the formation of Fbh1 foci in the absence of Rad51 after DNA damage (Fig 7C). This result suggests that Dbl2 recruits Fbh1 to DNA lesions before Rad51-ssDNA filament and JM formation. This feature may reflect a type of “fail-safe” mechanism, in which the repair machinery is recruited even before it is needed. This appears to be the case for some other events in meiosis. For example, in some species, such as *S*. *cerevisiae*, the Mre11-Rad50-Nbs1 (MRN) complex is required for meiotic DSB formation even though its catalytic activity is apparently needed only after DSB formation, *i*.*e*., for the processing of DNA ends to allow Rad51-ssDNA filament formation [67]. If Fbh1 is recruited to most meiotic DSBs before JM formation, it may play a more prominent role in meiotic DSB repair than discussed above: it may aid metabolism of JMs other than the rare (few per cell) persistent JMs detected in *dbl2Δ* mutants (Figs 4B, 4C and S4).

Purified Fbh1 can remove Rad51 from Rad51-ssDNA filaments and has DNA helicase activity [22]. Therefore, both Rad51 foci and JMs should accumulate in *dbl2Δ* and *fbh1Δ* mutants, as observed (Table 2; Figs 4B, 4C and S4). This is not the case for other *S*. *pombe* helicase mutants, *fml1Δ* and *fml2Δ*, or a chromatin remodeling mutant, *rdh54Δ* (Table 2). Thus, Fbh1 has a special role, which may be related to its having a second activity, ubiquitin ligase as part of the SCF complex [68]. We cannot distinguish whether the phenotypes observed here are due to loss of the helicase or the ligase activity, or both, but the accumulation of JMs (Figs 4B, 4C and S4) suggests that the Fbh1 helicase is important for meiotic joint molecule metabolism. The ligase activity may also be important to remove Rad51, but this action might be a result of the helicase alone.

### Multiple means for meiotic DNA joint molecule processing

Previous work has shown that during meiosis JMs can be processed in a variety of ways into chromosomes suitable for segregation. In *S*. *cerevisiae* at least six mechanisms have been shown or inferred to convert JMs into recombinant chromosomes [69,70] (and references therein). In contrast, in *S*. *pombe* only one mechanism has been described–the formation of HJs and their resolution nearly exclusively by the Mus81-Eme1 resolvase [27–29,44,71]. Here, we propose an additional mechanism–Fbh1, recruited by Dbl2, acts on D-loops to reverse them or to convert them into HJs suitable for Mus81-Eme1 resolution. This action is important, for in the absence of Dbl2 viable spore yields are reduced by a factor of 13 (Table 1) and chromosomes missegregate in up to half of the cells (Figs 1, 2 and 3). There is little effect on recombinant frequencies, however, because these JMs are rare (Figs 4B and S4) and, we propose, many of these JMs are intersister, which cannot produce recombinants. Thus, studies of recombination have overlooked the important function of Dbl2 and Fbh1 for meiotic chromosome segregation.

### Conservation of Dbl2 function

Dbl2 is predicted to encode a 78 kDa protein with a domain of unknown function (DUF2439) at the N-terminus. Our bioinformatic searches revealed that within the DUF2439 region Dbl2 is highly conserved in fungi, animals and plants. Orthologs can be detected in *Saccharomyces cerevisiae* (Mte1, YGR042W), *Homo sapiens* (ZGRF1) and *Arabidopsis thaliana* (AT4G10890) (S8 Fig). The only other sequence family that is significantly related to the DUF2439 domain is the one including Rdh54 (*S*. *cerevisiae*, *S*. *pombe*) and RAD54B (*H*. *sapiens*) (S8 Fig). Interestingly, some proteins of both sequence families contain an AAA+ ATPase domain and/or a helicase domain (ZGRF1, Rdh54, RAD54B) at their C-termini. The C-terminal part of Dbl2 is conserved only within the *Schizosaccharomyces* species; it is enriched for polar residues and is predicted to be highly disordered and thus unlikely to form an active helicase. However, Dbl2 is required for targeting helicases Fml1 and Fbh1 to DSBs (this work and [31]). Similarly, recent studies in *S*. *cerevisiae* showed that Mte1 is required for localization of Mph1 helicase at sites of DNA damage and regulates Mph1 activity [72–74]. Thus, we speculate that Dbl2 acts as an adaptor or recruiter for helicases Fml1 and Fbh1 and perhaps for other proteins. The mechanism may be similar to that of other adaptor proteins containing disordered C-terminal regions such as endocytic adaptor proteins Epsin1 and AP180 and Atg13 adaptor, which controls the initiation of autophagy [75,76].

What is the role of the DUF2439 domain? Surprisingly, the N-terminal truncation that removes the DUF2439 domain had no effect on either Dbl2 focus formation at DSBs or on its ability to confer camptothecin-resistance [31]. Similarly, deletion of the budding yeast *rdh54* confers sensitivity to MMS but N-terminal truncations of Rdh54 that remove or truncate the DUF2439 domain do not affect MMS sensitivity [77]. Interestingly, these Rdh54 truncation mutants lost their ability to interact with Rad51 and had impaired ability to dissociate Rad51-DNA complexes [77–79]. The reason why the DUF2439 domain is important for Rad51 interaction but not for resistance to camptothecin or MMS is not known, but one possible explanation is that the role of Dbl2 and Rdh54 in repair of MMS- or camptothecin-induced DNA damage may not be through Rad51. The N-terminal domain of human RAD54B which includes the DUF2439 domain binds to branched DNA substrates and interacts with both RAD51 and DMC1 [80]. Similarly as in Rdh54 and RAD54B, the DUF2439 domain of Dbl2 may mediate interaction with Rad51. This would allow Dbl2 to bring Fml1 and Fbh1 helicases directly to Rad51 nucleoprotein filaments. Consistent with this notion is our finding that Dbl2 interacts with Rad51 and weakly with Fml1 in yeast two-hybrid assays (S9 Fig). Interestingly, our observation that the Fbh1 foci are formed in the absence of Rad51 (Fig 7C) indicates that, to some extent, Dbl2 is able to promote Fbh1 focus-formation at meiotic DSBs independently of Rad51. We observed no interaction between Dbl2 and Fbh1 in yeast two-hybrid assays (S9 Fig). These results do not, however, exclude the possibility that Dbl2 interacts directly with Fbh1.

Both Dbl2 and Fbh1 are evolutionarily conserved proteins present from yeast to humans, suggesting that our results may apply widely. A notable exception is the absence of an Fbh1 homolog in the budding yeast *S*. *cerevisiae*. However, it has been proposed that *S*. *cerevisiae* Srs2 helicase is a functional counterpart of Fbh1 [81]. The Dbl2 and Fbh1 proteins, like other proteins central to DNA repair, are widely conserved and play crucial roles in maintaining cell viability, genome integrity, and high fertility, making their further study important.

## Materials and Methods

### Strains, growth media and general methods

The genotypes of the yeast strains used in this study are listed in the S1 Table. Standard media (rich YES and appropriately supplemented minimal EMM2 and sporulation SPA) were used to maintain, grow and mate *S*. *pombe* strains [39,82–84]. To induce mating and meiosis (Figs 1, 2, 3, 6 and 7 and Table 2), cells were grown in liquid YES to mid-log phase at 32°C, washed three times with water, mixed (for heterothallic matings), transferred to EMM2-NH_4_Cl plates, and incubated at 25°C for 10–17 hr before examination [85]. DNA was extracted from meiotically induced cells and analyzed as described [86]. *S*. *pombe* was transformed using the lithium acetate method and genes deleted as described [87]. The immunostaining and microscopy used to analyze chromosome segregation and subcellular localization of Rec8 and Rad51 in *S*. *pombe* cells were performed as described [7]. Subcellular localization of Rad51 was determined using anti-Rhp51 polyclonal antibody (Cosmo Bio) diluted 1:500. No foci were detected in *rad51Δ* mutant cells, indicating that in our assays anti-Rhp51 antibody specifically detected Rad51 (S10 Fig and Table 2). Live-cell imaging and spore viability determinations were performed as described [88]. Viable spore yields and recombinant frequencies (Table 1) were determined as described [89].

### Plasmid loss assay

*fbh1∆* (JG17775) and *fbh1∆ dbl2∆* (JG17777) cells harboring plasmid pMW651 (expressing Fbh1-YFP and containing the *LEU2*^*+*^ marker, which complements the *leu1-32* mutation in the parental strains) were grown in selective EMM medium lacking leucine at 32°C to mid-log phase. The cells were washed with water and resuspended in nonselective YES liquid medium, incubated at 32°C for 18 hr (7 generations) and then plated on YES plates. Colonies were formed after 3 days at 32°C and replica-plated onto selective plates (EMM lacking leucine). The percentage of plasmid loss per generation was determined as described by Osman et al. [90].

### Yeast two-hybrid analysis

Full-length coding regions for Dbl2, Fbh1, Fml1 and Rad51 were amplified from meiotic cDNA with primers that added a 5' *Sfi*I and 3' *Sma*I or *Bam*HI (Fbh1 only) restriction sites and then cloned into plasmid pGBKT7 (Clontech) for GAL4-DNA binding-domain bait constructs, or into plasmid pGADT7 (Clontech) for GAL4 activation domain prey constructs. Primers used were 5'-AAAAGGCCATGGAGGCCATGGATACAAGTTCCAATGTTTTTCATTATC-3' and 5'-AAAACCCGGGTCAAATAAAGTCACCATCTTCGTCCGAATC-3' for Dbl2, 5'-AAAAGGCCATGGAGGCCATGAGTGCTCAACATTTACATAGCTGCAAAT-3' and 5’-AAAAGGATCCCTACTGATCATGTACAGCAAACAATTGATTTTCAATAAATAGCATCGATCTTTTAAGCCG-3' for Fbh1, 5'-AAAAGGCCATGGAGGCCATGTCCGATGATTCTTTTAGTAGTGATGAAG-3' and 5'-AAAACCCGGGCTAAATCAGCATTCCTTTCATACGTTTCCTTTTC-3' for Fml1, and 5'-AAAAGGCCATGGAGGCCATGGCAGATACAGAGGTGGAAATGCAAGTTAG-3' and 5'-AAAACCCGGGTTAGACAGGTGCGATAATTTCCTTGGGATCACCAACACC-3' for Rad51. Bait and prey plasmids were introduced into *S*. *cerevisiae* strain PJ69-4a (Clontech) by standard lithium acetate-mediated transformation. Transformants were tested for bait-prey interaction by spotting onto SD minimal media lacking appropriate amino acids according to the manufacturer's instructions. We performed at least two independent transformations and growth tests.

### Bioinformatics analysis

#### The DUF2439 domain is conserved within the Dbl2/ZGRF1 protein family in fungi, metazoa and plants

In a series of NCBI-BLAST searches [91] within the NCBI non-redundant protein database using *S*. *pombe* Dbl2 as starting query and applying significant e-value thresholds (<0.001), we identified an N-terminally located region (Dbl2 amino acids 7 to 77) conserved in fungi, animals and plants, that is also described as the domain of unknown function DUF2439 in the Pfam database [92]. Orthologues include *S*. *cerevisiae* YGR042W (= Mte1) and human ZGRF1, and reciprocal NCBI-Blast searches in the *S*. *pombe* proteome with the respective orthologous regions significantly yielded only Dbl2. The animal ZGRF1 protein family is extended, relative to yeast proteins, by one GRF zinc finger region and two AAA+ ATPase domains at the C-terminal half.

#### The Rdh54/RAD54B protein family shares the DUF2439 domain

To find distantly related DUF2439-containing proteins, we performed iterative NCBI-PSIBLAST searches. For 16 out of 17 queries, the NCBI-PSIBLAST search converged before it reached the specified threshold of 20 iterations. Other than the Dbl2 and ZGRF1 proteins, all significant hits were to an unidentified conserved domain in the Rdh54/RAD54b family. For example, *S*. *pombe* Dbl2 (residues 3–85) found *Cryptococcus gattii* Rad54b in iteration 3 (e-value of 2 x 10^−4^), fission yeast Rdh54 in iteration 4 (e-value of 3 x 10^−7^), and human RAD54b in iteration 6 (e-value of 1 x 10^−6^). The relationship was confirmed in a reciprocal search with *S*. *pombe* Rdh54 (residues 49–128), which identified Dbl2 in iteration 6 (e-value of 6 x 10^−4^) and human ZGRF1 in iteration 4 (e-value of 8 x 10^−5^). In an independent approach, using hmmsearch with the Pfam DUF2439 hidden Markov model [93], Rdh54 and Rad54b proteins were among the significant hits, including *S*. *pombe* Rdh54 (e-value of 2.2 x 10^−5^) and *Dictyostelium fasciculatum* RAD54b (e-value of 3.1 x 10^−5^). Whereas Rad54 and RAD54b protein families share the same overall domain architecture, including their DEAD-like helicase domain, the N-terminal region is specific to each protein. In human RAD54b, the N-terminal region has been reported to bind to Rad51 and Dmc1 and branched DNA structures [80].

## Supporting Information

S1 Fig Lagging chromatin in *dbl2Δ* mutant cells contains telomeric but not centromeric regions. The localization of the *sod2* (telomeric) and the *cen2* (centromeric) loci marked by LacI-GFP was scored in 100 *h*^*90*^ *dbl2Δ* (JG17271 and JG17130, respectively) anaphase I cells showing lagging chromatin and in 100 *h*^*90*^ wild-type anaphase I cells (JG12619 and JG12618, respectively). The strains were fixed and immunostained for tubulin and GFP; DNA was visualized by Hoechst staining.
(TIF)

S2 Fig Most of the Rec8-GFP is removed from chromatin during anaphase I in both wild-type and *dbl2Δ* mutant cells. Strains were sporulated on SPA and at 10–17 hr fixed and immunostained for tubulin and GFP; DNA was visualized by Hoechst staining. Representative images show the Rec8-GFP signal during anaphase I in wild type (JG13990) and *dbl2Δ* (JG17236) cells as well as in a mononucleate *dbl2Δ* zygote with two spindles.
(TIF)

S3 Fig Asci with abnormal size and number of spores are formed in *dbl2Δ* mutants. Sporulating wild-type (JG11355) and *dbl2Δ* cells (JG17146) were fixed and analyzed by DIC microscopy.
(TIF)

S4 Fig Holliday junctions are formed and repaired similarly in wild-type and *dbl2Δ* mutants, but Rec12-dependent joint molecules persist in *dbl2Δ* late meiosis–analysis at the *ade6-3049* DSB hotspot. **(A)** As shown in Fig 4A, DSBs are formed and repaired in a similar manner in strains GP6656 (*dbl2*^*+*^) and GP8664 (*dbl2Δ*). The results are from two independent experiments from which extracted DNA was digested with NotI and analyzed by pulsed-field gel electrophoresis and Southern blot hybridization using a probe at the left end of the 501 kb *Not*I fragment J (band at the top of the gel) [42]. The fraction of total DNA broken at *mbs1* and at *mbs2* (indicated by arrows on the right) at the indicated time for each strain is shown below each blot. **(B)** Strains GP6656 (*dbl2*^*+*^), GP8664 (*dbl2Δ*), and GP8836 (*rec12Δ dbl2Δ*) were induced and their DNA analyzed as in Fig 4B and 4C for joint DNA molecules. Southern blots of DNA extracted at the indicated times after meiotic induction were hybridized with a radioactive probe (~1 kb long) near the *ade6-3049* DSB hotspot on the 11.8 kb *Bsr*GI fragment or near the *mbs1* hotspot on the 10.5 kb *Bsr*GI fragment [1]. Black arrowheads indicate Y-shaped replication (0–3 hr) and recombination intermediates (4–5 hr); white arrowsheads indicate Holliday junctions at 4 and 5 hr. Persistent joint molecules seen at 6, 7, and 8 hr are X-shaped (white arrows) or Y-shaped (black arrows). These joint molecules at the *ade6-3049* hotspot persist in *dbl2Δ* but not in *dbl2*^*+*^ and are Rec12-dependent. **(C)** Analysis of DNA at both hotspots from independent inductions. **(D)** Quantification of data for *ade6-3049* from blots in S4 Fig, panels A and B, and additional experiments. See S2 and S3 Tables for individual data at each hotspot.
(TIF)

S5 Fig Synthetic growth defect of *dbl2Δ eme1Δ* double mutant strain. **(A)** *dbl2Δ* (JG17148) and *eme1Δ* (JG17465) strains were crossed, and asci were subjected to tetrad analysis. From one such representative tetrad, growth of the four spore colonies with the indicated genotypes is shown. **(B)** 10-fold dilutions of wild-type strain (JG17894), *dbl2Δ* mutant strain (JG17895), *eme1Δ* mutant strain (JG17896) and *dbl2Δ eme1Δ* double mutant strain (JG17897) were spotted on YES plates and incubated at 32°C for 3 days. Three independent cultures of slow-growing strain JG17897 (*dbl2Δ eme1Δ*) were tested.
(TIF)

S6 Fig Dbl2 is required for efficient targeting of Fbh1 to DNA lesions induced by MMS. **(A** and **B)** *S*. *pombe* strains expressing YFP-Fbh1 from plasmid pMW651 and carrying *fbh1Δ* (JG17775) or *fbh1Δ dbl2Δ* (JG17777) mutations growing in EMM2 medium without leucine at 32°C were treated with MMS (0.025%) for 4 hr and fixed; DNA was visualized with DAPI. The *dbl2Δ* mutant showed significantly fewer number of YFP-Fbh1 foci in G2 cells compared to those in *dbl2*^*+*^. The values reported are means of three independent experiments ± SEM. In each experiment 200 G2 cells were scored.
(TIF)

S7 Fig Rad52-mCherry foci, representing sites of active DNA repair, are not reduced in *dbl2Δ* mutant cells. *S*. *pombe* wild-type strain (JG17460) and *dbl2Δ* mutant strain (JG17510) expressing Rad52-mCherry from the native promoter were grown to exponential phase in liquid YES medium, treated with either 5 μM CPT **(A)** or 0.025% MMS **(B)** for 4 hr, fixed, and examined by fluorescence microscopy; DNA was visualized with DAPI. Data are the means of three independent experiments ± SEM. Rad52-mCherry foci were scored in three sets of 200 G2 cells.
(TIF)

S8 Fig The DUF2439 family is present in the Dbl2/Zgrf1 and Rdh54/RAD54B protein families. Multiple alignment of the indicated proteins from various species was performed with MAFFT (version 6, L-INS-I method) [2] and visualized in Jalview [3], using the ClustalX colouring profile. The sequence identifiers from the NCBI protein database are given in parentheses. The numbers of the first and last residues flank the region aligned.
(TIF)

S9 Fig Dbl2 interacts with Rad51 and Fml1 in yeast two-hybrid assay. Strains expressing Dbl2 fused to the GAL4 transcription activation domain and Rad51, Fbh1 or Fml1 fused to the GAL4 DNA-binding domain were grown on SD plates lacking tryptophan and leucine (SD-L,W) and then spotted at 5-fold serial dilutions on SD plates lacking tryptophan and leucine (SD-L,W) or SD plates lacking tryptophan, leucine and histidine (SD-L,W,H) or SD plates lacking tryptophan, leucine and adenine (SD-L,W,A). The empty vectors pGADT7 and pGBKT7 containing GAL4 transcription activation domain and GAL4 DNA-binding domain, respectively were used as negative controls. Growth on plates without histidine or without adenine indicates interaction between the fusion proteins [4].
(TIF)

S10 Fig Anti-Rhp51 antibody detects foci in wild-type but not *rad51Δ* zygotes. To test the specificity of anti-Rhp51 antibody, we analyzed subcellular localization of Rad51 using anti-Rhp51 polyclonal antibody (Cosmo Bio) diluted 1:500 in wild-type (JG11355) and *rad51Δ* (JG17993, JG17540) prophase I cells. Cells were mated on SPA sporulation agar and at 10–17 hr fixed and immunostained for tubulin and Rad51, and examined by fluorescence microscopy; DNA was visualized by Hoechst staining.
(TIF)

S1 Table *S*. *pombe* strains. (DOCX)

S2 Table Holliday junctions are formed and repaired similarly in wild-type and *dbl2Δ* mutant, but Rec12-dependent joint molecules persist in *dbl2Δ* late meiosis–analysis at the *mbs1* DSB hotspot. (DOCX)

S3 Table Holliday junctions are formed and repaired similarly in wild-type and *dbl2Δ* mutant, but Rec12-dependent joint molecules persist in *dbl2Δ* late meiosis–analysis at the *ade6-3049* DSB hotspot. (DOCX)

S4 Table Dbl2 is required for efficient targeting of Fbh1 to DNA lesions induced by CPT. (DOCX)

S5 Table Dbl2 is required for efficient targeting of Fbh1 to DNA lesions induced by deletion of genes involved in homologous recombination. (DOCX)

S6 Table Dbl2-YFP forms foci independently of Fbh1 in cells with DNA lesions induced by CPT or MMS. (DOCX)

S1 References Supporting Information References. (DOCX)
