# Supplementary figures and images for: Dbl2 Regulates Rad51 and DNA Joint Molecule Metabolism to Ensure Proper Meiotic Chromosome Segregation
Source: PLoS Genet. 2016 Jun 15;12(6):e1006102. doi: 10.1371/journal.pgen.1006102 (PMC4909299; doi:10.1371/journal.pgen.1006102)

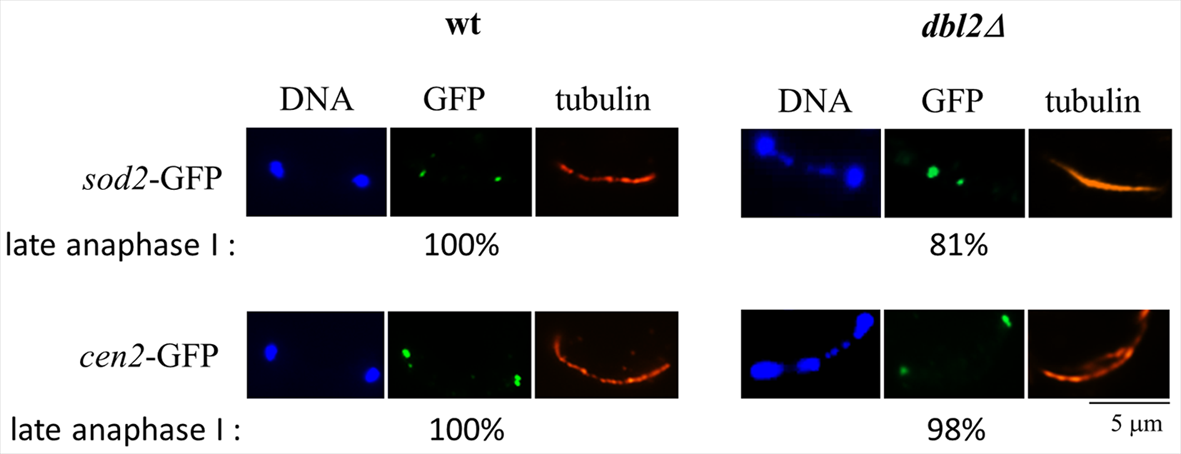

Supplement: S1 Fig — The localization of the sod2 (telomeric) and the cen2 (centromeric) loci marked by LacI-GFP was scored in 100 h90 dbl2Δ (JG17271 and JG17130, respectively) anaphase I cells showing lagging chromatin and in 100 h90 wild-type anaphase I cells (JG12619 and JG12618, respectively). The strains were fixed and immunostained for tubulin and GFP; DNA was visualized by Hoechst staining. (TIF) [file pgen.1006102.s001.tif]

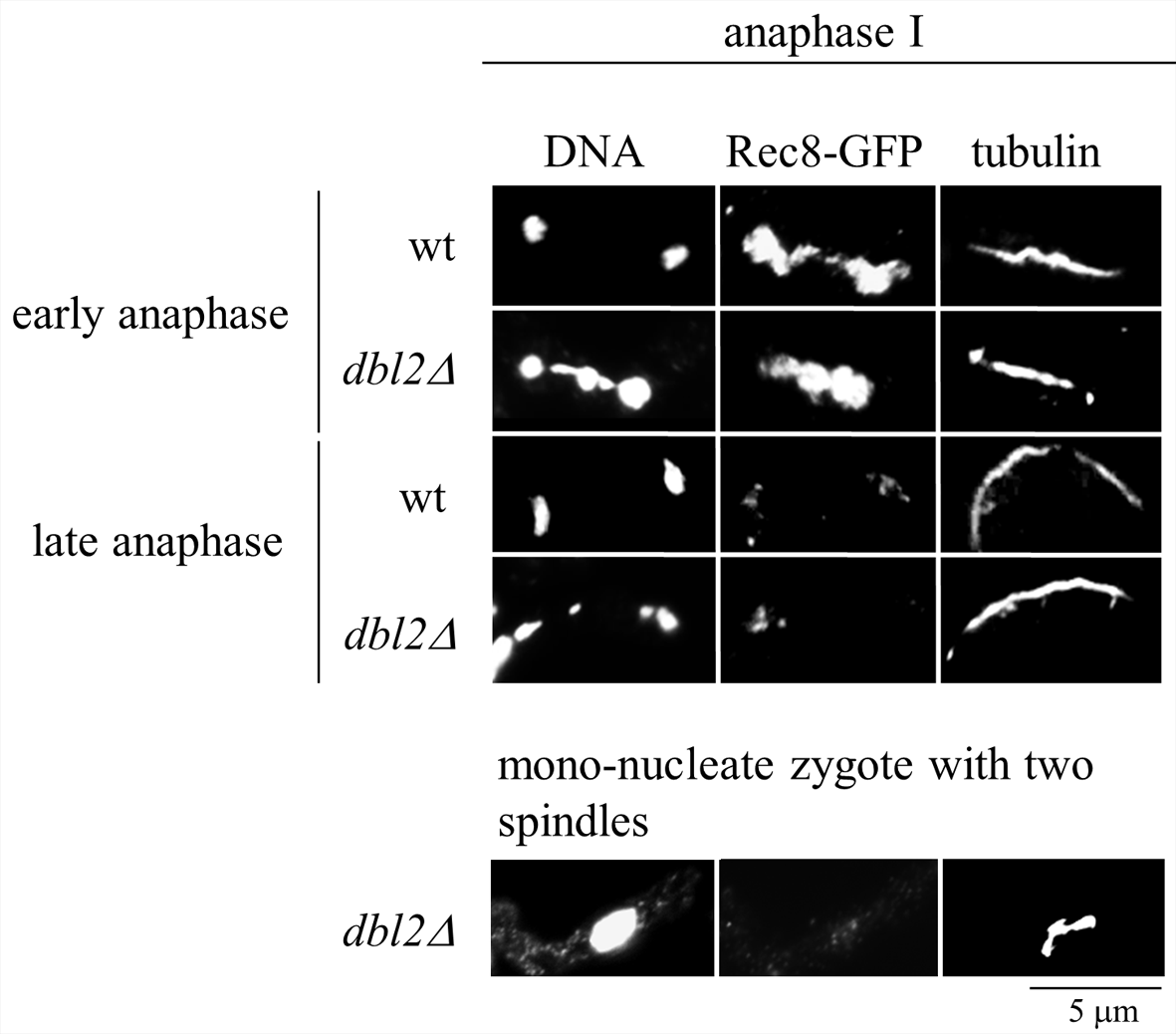

Supplement: S2 Fig — Strains were sporulated on SPA and at 10–17 hr fixed and immunostained for tubulin and GFP; DNA was visualized by Hoechst staining. Representative images show the Rec8-GFP signal during anaphase I in wild type (JG13990) and dbl2Δ (JG17236) cells as well as in a mononucleate dbl2Δ zygote with two spindles. (TIF) [file pgen.1006102.s002.tif]

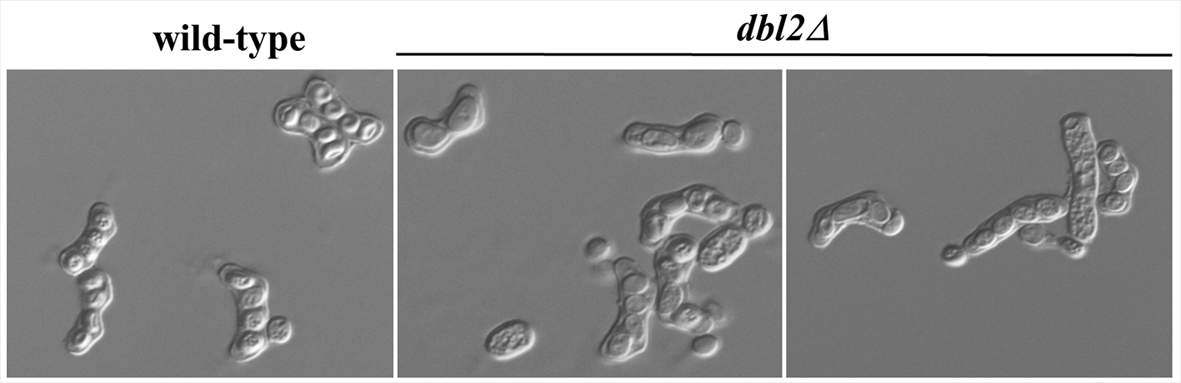

Supplement: S3 Fig — Sporulating wild-type (JG11355) and dbl2Δ cells (JG17146) were fixed and analyzed by DIC microscopy. (TIF) [file pgen.1006102.s003.tif]

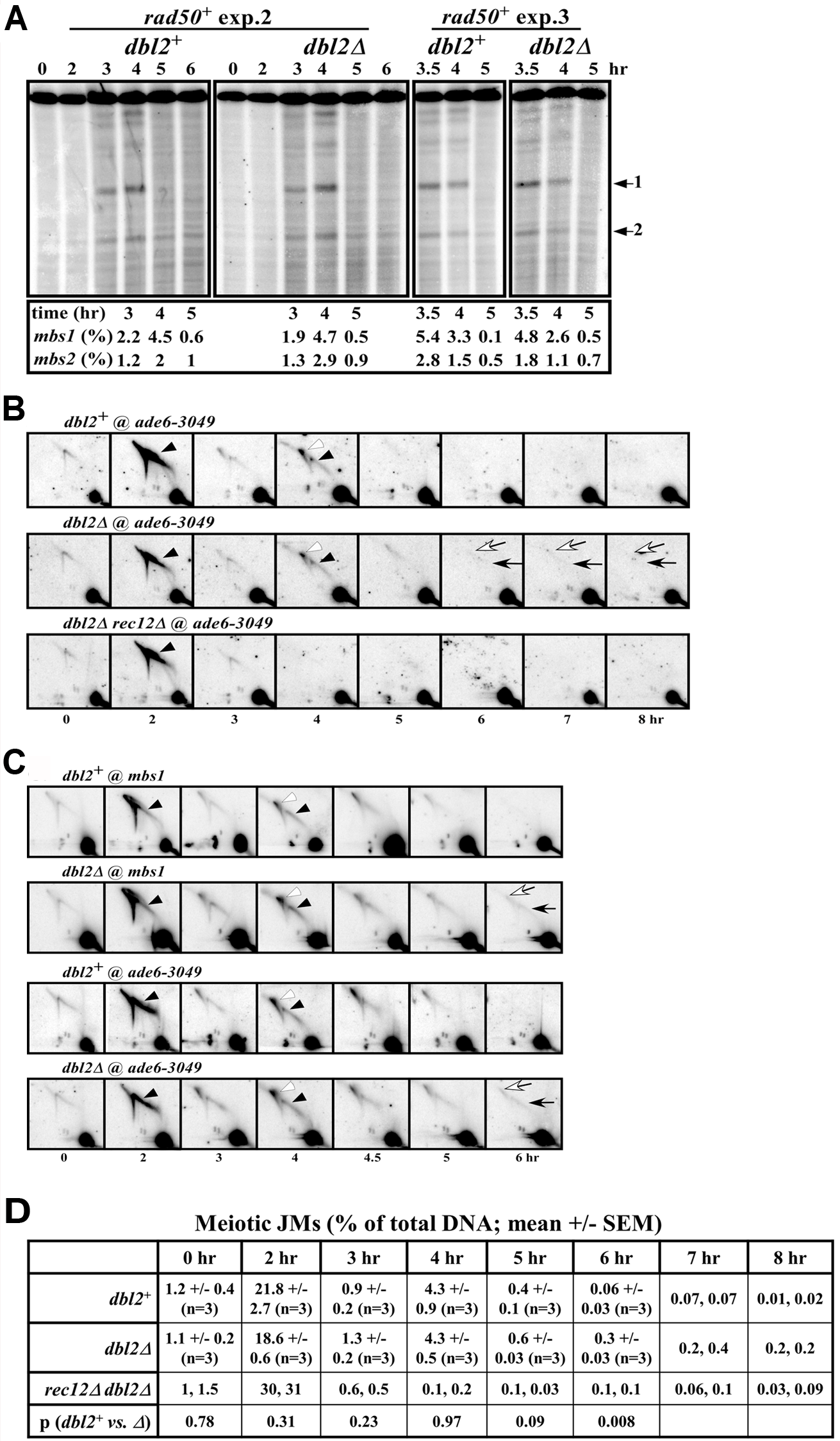

Supplement: S4 Fig — (A) As shown in Fig 4A, DSBs are formed and repaired in a similar manner in strains GP6656 (dbl2+) and GP8664 (dbl2Δ). The results are from two independent experiments from which extracted DNA was digested with NotI and analyzed by pulsed-field gel electrophoresis and Southern blot hybridization using a probe at the left end of the 501 kb NotI fragment J (band at the top of the gel) [42]. The fraction of total DNA broken at mbs1 and at mbs2 (indicated by arrows on the right) at the indicated time for each strain is shown below each blot. (B) Strains GP6656 (dbl2+), GP8664 (dbl2Δ), and GP8836 (rec12Δ dbl2Δ) were induced and their DNA analyzed as in Fig 4B and 4C for joint DNA molecules. Southern blots of DNA extracted at the indicated times after meiotic induction were hybridized with a radioactive probe (~1 kb long) near the ade6-3049 DSB hotspot on the 11.8 kb BsrGI fragment or near the mbs1 hotspot on the 10.5 kb BsrGI fragment [1]. Black arrowheads indicate Y-shaped replication (0–3 hr) and recombination intermediates (4–5 hr); white arrowsheads indicate Holliday junctions at 4 and 5 hr. Persistent joint molecules seen at 6, 7, and 8 hr are X-shaped (white arrows) or Y-shaped (black arrows). These joint molecules at the ade6-3049 hotspot persist in dbl2Δ but not in dbl2+ and are Rec12-dependent. (C) Analysis of DNA at both hotspots from independent inductions. (D) Quantification of data for ade6-3049 from blots in S4 Fig, panels A and B, and additional experiments. See S2 and S3 Tables for individual data at each hotspot. (TIF) [file pgen.1006102.s004.tif]

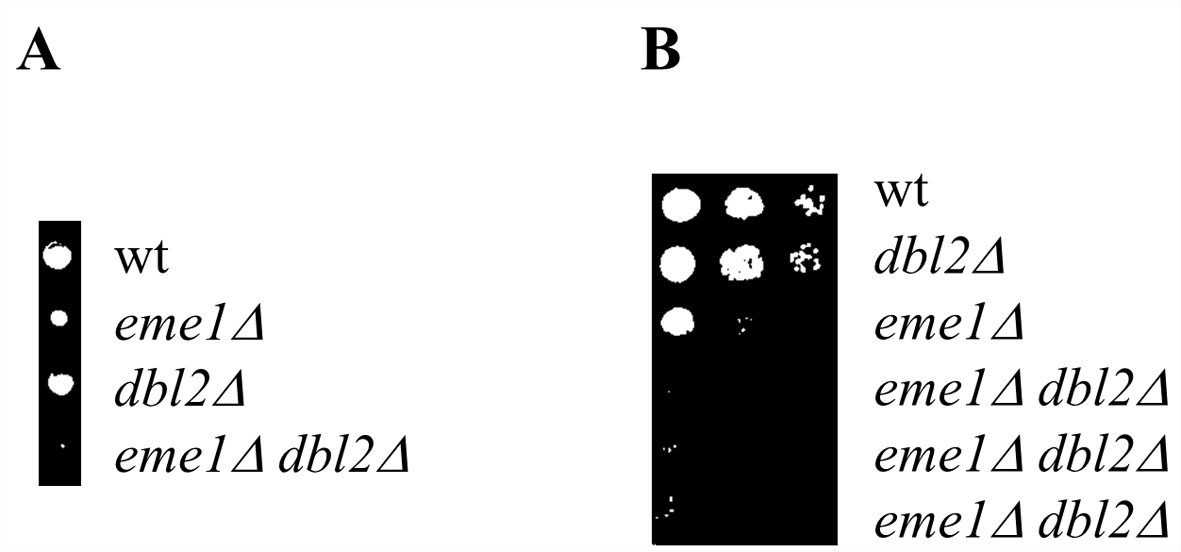

Supplement: S5 Fig — (A) dbl2Δ (JG17148) and eme1Δ (JG17465) strains were crossed, and asci were subjected to tetrad analysis. From one such representative tetrad, growth of the four spore colonies with the indicated genotypes is shown. (B) 10-fold dilutions of wild-type strain (JG17894), dbl2Δ mutant strain (JG17895), eme1Δ mutant strain (JG17896) and dbl2Δ eme1Δ double mutant strain (JG17897) were spotted on YES plates and incubated at 32°C for 3 days. Three independent cultures of slow-growing strain JG17897 (dbl2Δ eme1Δ) were tested. (TIF) [file pgen.1006102.s005.tif]

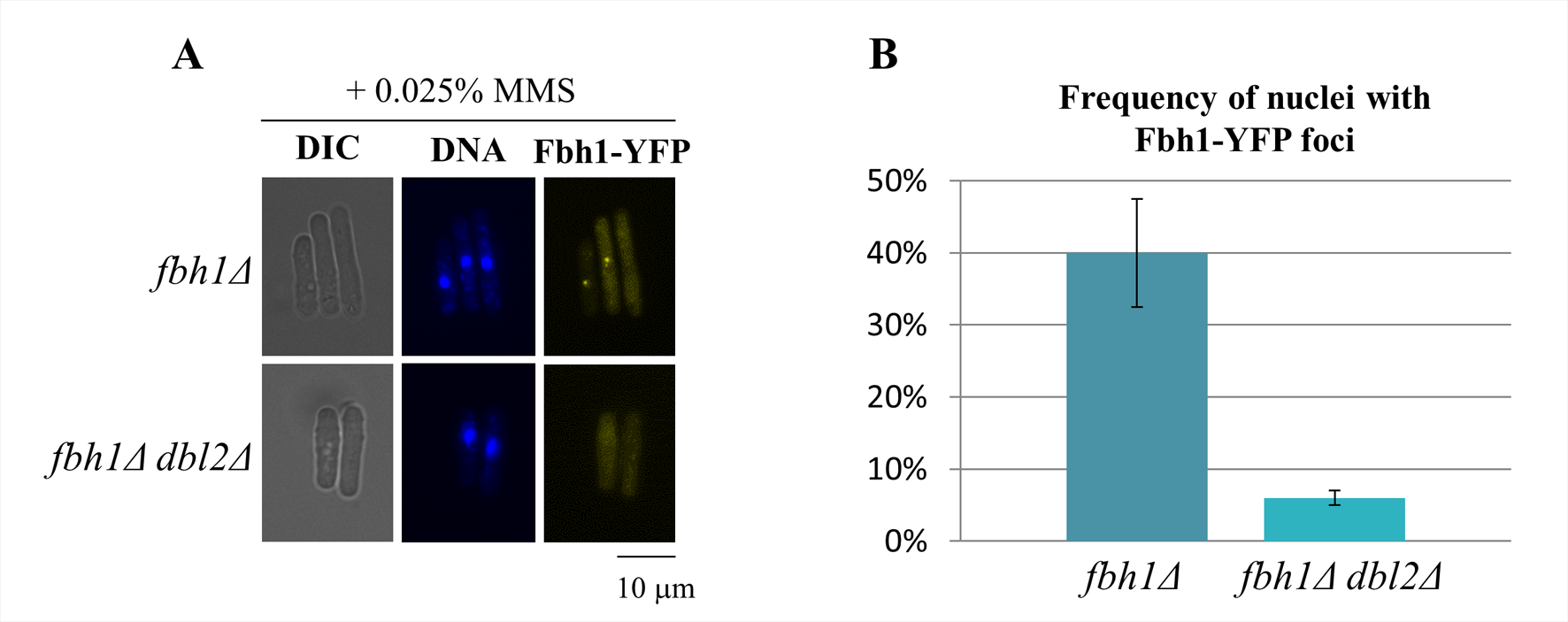

Supplement: S6 Fig — (A and B) S. pombe strains expressing YFP-Fbh1 from plasmid pMW651 and carrying fbh1Δ (JG17775) or fbh1Δ dbl2Δ (JG17777) mutations growing in EMM2 medium without leucine at 32°C were treated with MMS (0.025%) for 4 hr and fixed; DNA was visualized with DAPI. The dbl2Δ mutant showed significantly fewer number of YFP-Fbh1 foci in G2 cells compared to those in dbl2+. The values reported are means of three independent experiments ± SEM. In each experiment 200 G2 cells were scored. (TIF) [file pgen.1006102.s006.tif]

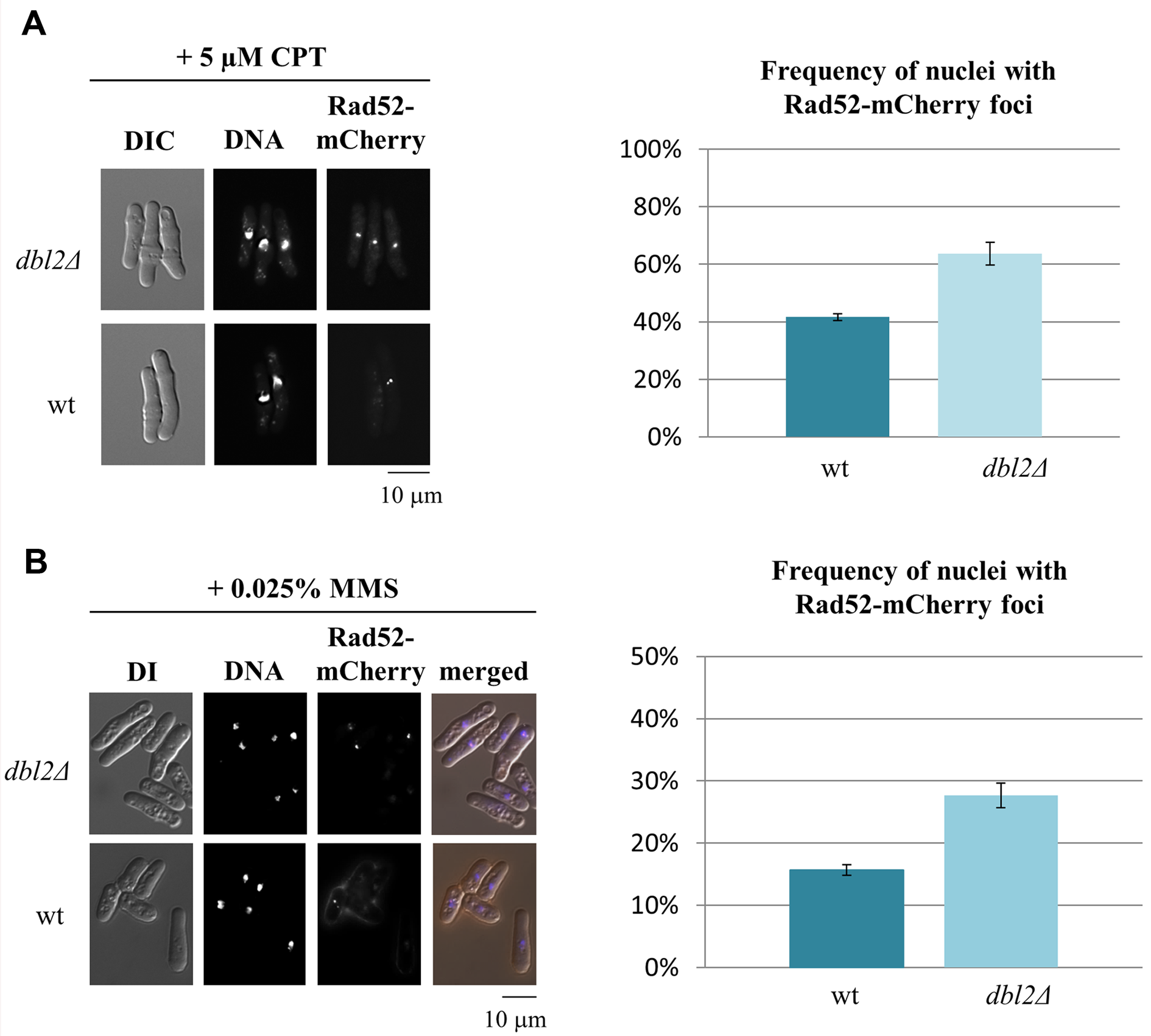

Supplement: S7 Fig — S. pombe wild-type strain (JG17460) and dbl2Δ mutant strain (JG17510) expressing Rad52-mCherry from the native promoter were grown to exponential phase in liquid YES medium, treated with either 5 μM CPT (A) or 0.025% MMS (B) for 4 hr, fixed, and examined by fluorescence microscopy; DNA was visualized with DAPI. Data are the means of three independent experiments ± SEM. Rad52-mCherry foci were scored in three sets of 200 G2 cells. (TIF) [file pgen.1006102.s007.tif]

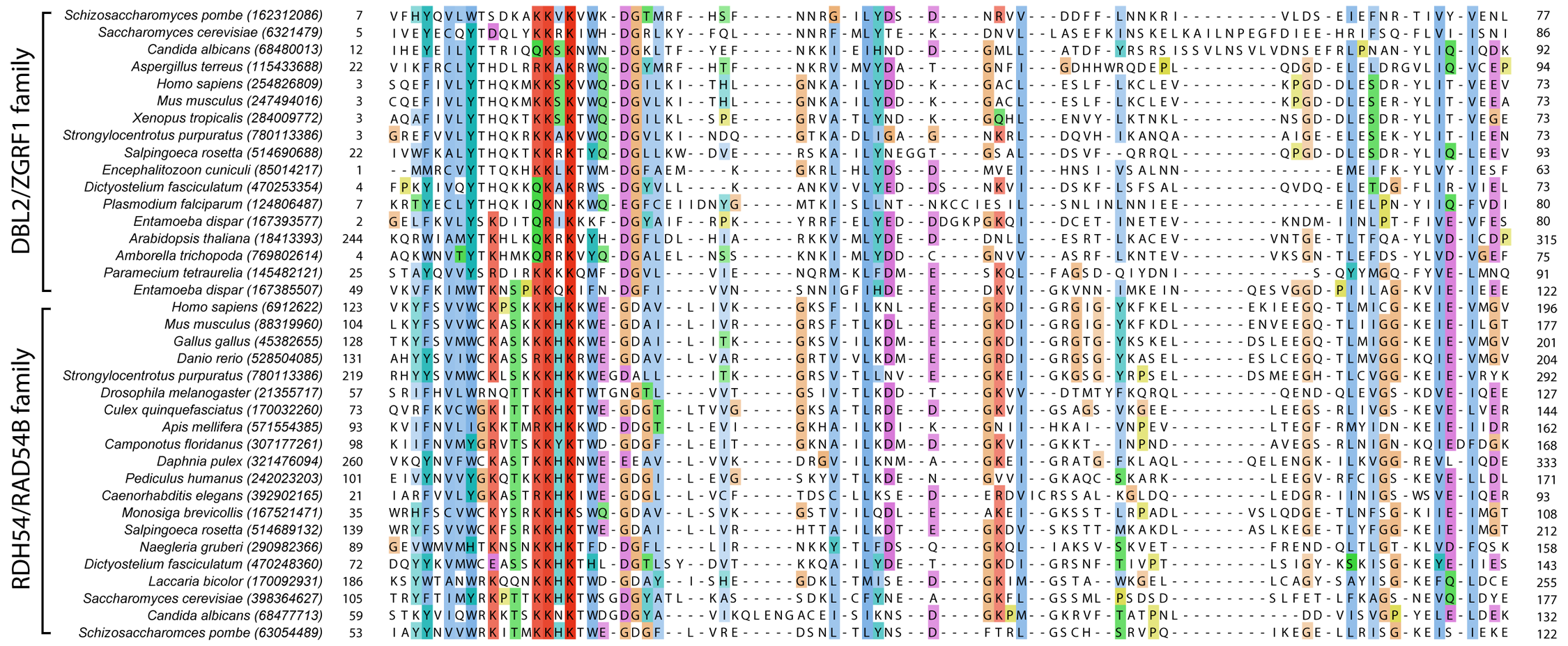

Supplement: S8 Fig — Multiple alignment of the indicated proteins from various species was performed with MAFFT (version 6, L-INS-I method) [2] and visualized in Jalview [3], using the ClustalX colouring profile. The sequence identifiers from the NCBI protein database are given in parentheses. The numbers of the first and last residues flank the region aligned. (TIF) [file pgen.1006102.s008.tif]

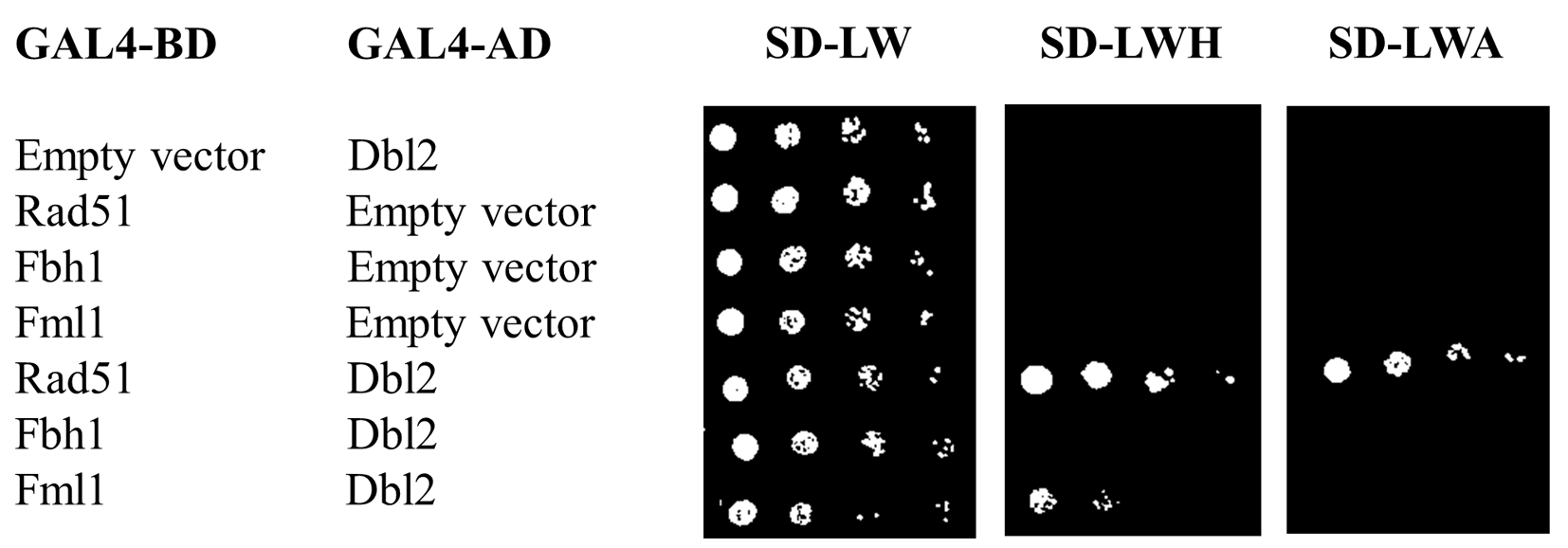

Supplement: S9 Fig — Strains expressing Dbl2 fused to the GAL4 transcription activation domain and Rad51, Fbh1 or Fml1 fused to the GAL4 DNA-binding domain were grown on SD plates lacking tryptophan and leucine (SD-L,W) and then spotted at 5-fold serial dilutions on SD plates lacking tryptophan and leucine (SD-L,W) or SD plates lacking tryptophan, leucine and histidine (SD-L,W,H) or SD plates lacking tryptophan, leucine and adenine (SD-L,W,A). The empty vectors pGADT7 and pGBKT7 containing GAL4 transcription activation domain and GAL4 DNA-binding domain, respectively were used as negative controls. Growth on plates without histidine or without adenine indicates interaction between the fusion proteins [4]. (TIF) [file pgen.1006102.s009.tif]

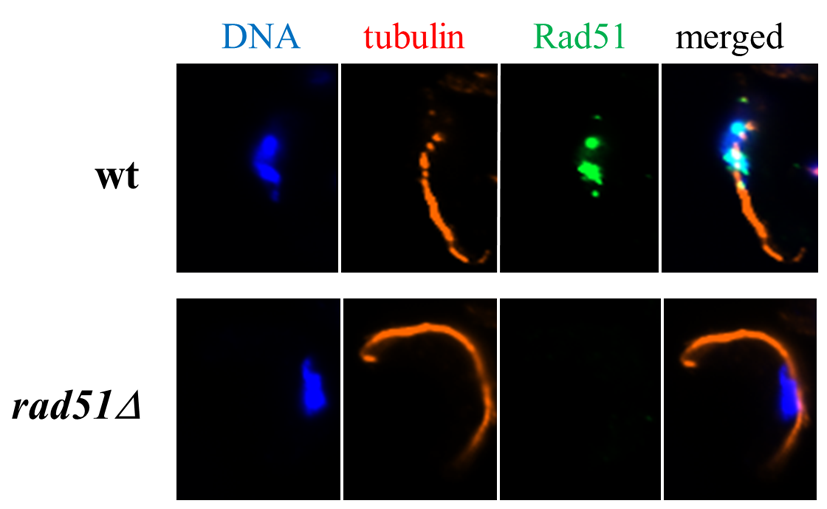

Supplement: S10 Fig — To test the specificity of anti-Rhp51 antibody, we analyzed subcellular localization of Rad51 using anti-Rhp51 polyclonal antibody (Cosmo Bio) diluted 1:500 in wild-type (JG11355) and rad51Δ (JG17993, JG17540) prophase I cells. Cells were mated on SPA sporulation agar and at 10–17 hr fixed and immunostained for tubulin and Rad51, and examined by fluorescence microscopy; DNA was visualized by Hoechst staining. (TIF) [file pgen.1006102.s010.tif]
