# Supplementary material for: Dbl2 Regulates Rad51 and DNA Joint Molecule Metabolism to Ensure Proper Meiotic Chromosome Segregation
Source: PLoS Genet. 2016 Jun 15;12(6):e1006102. doi: 10.1371/journal.pgen.1006102 (PMC4909299; doi:10.1371/journal.pgen.1006102)
Supplement: S1 Table — (DOCX) [file pgen.1006102.s011.docx]

**Table S1. *S. pombe* strains**

| Strain | Genotype | Used in |
| --- | --- | --- |
| JG12618 | *h^90^ ade6-M216 leu1-32 lys1-131 ura4-D18 cen2(D107)[::kanr-ura4^+^-lacOp] his7^+^::lacI-GFP* | Figure 1, 2 |
| JG17130 | *h^90^ ade6-M216 leu1-32 lys1-131 ura4-D18 cen2(D107)[::kanr-ura4^+^-lacOp] his7^+^::lacI-GFP dbl2::natMX4* | Figure 1, 2 |
| JG15456 | *h^+^ ade6-210 leu1-32 lys1-131 ura4-D18 cen2(D107) [::kanr-ura4^+^-lacOp] his7^+^::lacI-GFP* | Figure 1, 3 |
| JG17208 | *h^+^ ade6-210 leu1-32 lys1-131 ura4-D18 cen2(D107) [::kanr-ura4^+^-lacOp] his7^+^::lacI-GFP dbl2::natMX4* | Figure 1, 3 |
| JG11318 | *h^-^ leu1-32 ura4-D18 ade6-M210* | Figure 1, 3 |
| JG17207 | *h^-^ leu1-32 ura4-D18 ade6-M210 dbl2::natMX4* | Figure 1, 3 |
| JG12269 | *h^-^ leu1-32 lys1-131 ura4-D18 cen2(D107) [::kanr-ura4^+^-lacOp] his7^+^::lacI-GFP sgo1::natMX4* | Figure 3 |
| JG11793 | *h^+^ lys1 his7 leu1 ura4 ade6-M210 sgo1::natMX4* | Figure 3 |
| JG17779 | *h^+^ cen2(D107) [::kanr-ura4^+^-lacOp] his7^+^::lacI-GFP dbl2::natMX4 sgo1*:: *hphMX4* | Figure 3 |
| JG17780 | *h^-^ leu1-32 ura4-D18 ade6-M210 dbl2::natMX4* *sgo1*:: *hphMX4* | Figure 3 |
| JG16917 | *h^90^ ade6-M210 leu1-32 ura4-D18 hht1-mRFP-his3^+^MX6 pcp1-GFP-kanMX6::leu1* | Figure 1, 2 |
| JG17116 | *h^90^ ade6-M210 leu1-32 ura4-D18 hht1-mRFP-his3^+^MX6 pcp1-GFP-kanMX6::leu1 dbl2::natMX4* | Figure 1, 2 |
| JG17159 | *h^90^ ade6-M210 leu1-32 ura4-D18 hht1-mRFP-his3^+^MX6 pcp1-GFP-kanMX6::leu1 rec12*::*hphMX4* | Figure 2 |
| JG17156 | *h^90^ ade6-M210 leu1-32 ura4-D18 hht1-mRFP-his3^+^MX6 pcp1-GFP-kanMX6::leu1 dbl2::natMX4 rec12*::*hphMX4* | Figure 2 |
| JG17351 | *h^90^  ade6-M216 leu1-32 lys1-131 ura4-D18 cen2(D107)[:: kanr-ura4^+^-lacOp] his7^+^::lacI-GFP* *rec12*::*hphMX4* | Figure 2 |
| JG17353 | *h^90^  ade6-M216 leu1-32 lys1-131 ura4-D18 cen2(D107)[:: kanr-ura4^+^-lacOp] his7^+^::lacI-GFP* *dbl2::natMX4 rec12*::*hphMX4* | Figure 2 |
| JG17453 | *h^90^ leu1-32 ura4D18 ade6-216 pRep41-RusA::LEU2^+^* | Figure 5 |
| JG17452 | *h^90^ leu1-32 ura4D18 ade6-216 pRep41-RusA-D70N::LEU2^+^* | Figure 5 |
| JG17451 | *h^90^ leu1-32 ura4D18 ade6-216 dbl2::natMX4 pRep41-RusA::LEU2^+^* | Figure 5 |
| JG17450 | *h^90^ leu1-32 ura4D18 ade6-216 dbl2::natMX4 pRep41-RusA-D70::LEU2^+^* | Figure 5 |
| JG17444 | *h^+^ ade6-M216 ura4-D18 leu1-32 eme1::kanMX4* *pRep41-RusA::LEU2^+^* | Figure 5 |
| JG17445 | *h^+^ ade6-M216 ura4-D18 leu1-32 eme1::kanMX4* *pRep41-RusA-D70::LEU2^+^* | Figure 5 |
| JG17448 | *h^+^ ade6-M210 ura4-D18 leu1-32 rqh1::kanMX4* *pRep41-RusA::LEU2^+^* | Figure 5 |
| JG17449 | *h^+^ ade6-M210 ura4-D18 leu1-32 rqh1::kanMX4* *pRep41-RusA-D70::LEU2^+^* | Figure 5 |
| JG17953 | *h^90^ ura4D18 ade6-216 pRep41::LEU2^+^ (omns)^a^* | Figure 5, 8 |
| JG17955 | *h^90^ leu1-32 ura4D18 ade6-216 dbl2::natMX4 pRep41::LEU2^+^ (omns)^a^* | Figure 5, 8 |
| JG17959 | *h^+^ leu1-32 eme1::kanMX4 pRep41::LEU2^+^ (omns)^a^* | Figure 5 |
| JG17957 | *h^+^ leu1-32 rqh1::kanMX4 pRep41::LEU2^+^ (omns)^a^* | Figure 5 |
| JG17944 | *h^90^ leu1-32 fbh1::kanMX4 pRep41::LEU2^+^ (omns)^a^* | Figure 5 |
| JG17945 | *h^90^ leu1-32 fbh1::kanMX4 pRep41-RusA::LEU2^+^ (omns)^a^* | Figure 5 |
| JG17947 | *h^90^ leu1-32 fbh1::kanMX4 pRep41-RusA-D70N::LEU2^+^ (omns)^a^* | Figure 5 |
| JG17827 | *h^90^ leu1-32 eme1::kanMX4 (omns)^a^* | Figure 5 |
| JG17949 | *h^90^ leu1-32 eme1::kanMX4 pRep41-RusA::LEU2^+^ (omns)^a^* | Figure 5 |
| JG17951 | *h^90^ leu1-32 eme1::kanMX4 pRep41-RusA-D70N::LEU2^+^ (omns)^a^* | Figure 5 |
| JG17146 | *h^90^ leu1-32 ura4D18 ade6-216 dbl2::natMX4* | Figure 5, 6, Table 2 |
| JG11355 | *h^90^ leu1-32 ura4D18 ade6-216* | Figure 5, 6, Table 2 |
| JG17784 | *h^90^ fml1::kanMX4 (omns)^a^* | Table 2 |
| JG17786 | *h^90^ fml1::kanMX4 dbl2::natMX4 (omns)^a^* | Table 2 |
| JG17788 | *h^90^ fml2::kanMX4 (omns)^a^* | Table 2 |
| JG17790 | *h^90^ fml2::kanMX4 dbl2::natMX4 (omns)^a^* | Table 2 |
| JG17815 | *h^-^ smt0 rad54::kanMX4 (omns)^a^* | Table 2 |
| JG17817 | *h^+^ rad54::kanMX4 (omns)^a^* | Table 2 |
| JG17821 | *h^-^ smt0 rad54::kanMX4 dbl2::natMX4 (omns)^a^* | Figure 6, Table 2 |
| JG17819 | *h^+^ rad54::kanMX4 dbl2::natMX4 (omns)^a^* | Figure 6, Table 2 |
| JG17540 | *h^+^ rad51::hphMX4 (omns)^a^* | Table 2 |
| JG17506 | *h^-^ leu1-32 ura4-D18 ade6-M210 rad51::hphMX4* | Table 2 |
| JG17542 | *h^+^ rad51::hphMX4 dbl2::natMX4 (omns)^a^* | Table 2 |
| JG17507 | *h^-^ leu1-32 ura4-D18 ade6-M210 rad51::hphMX4 dbl2::natMX4* | Table 2 |
| JG17756 | *h^+^ rad55::kanMX4 ade6-52 omns* | Table 2 |
| JG17755 | *h^-^ rad55::kanMX4 ade6-375 omns* | Table 2 |
| JG17757 | *h^+^ rad55::kanMX4 dbl2::natMX4 ade6-375 (omns)^a^* | Table 2 |
| JG17758 | *h^-^ rad55::kanMX4 dbl2::natMX4 ade6-52 (omns)^a^* | Table 2 |
| JG17749 | *h^+^ rad57::kanMX4 (omns)^a^* | Table 2 |
| JG17750 | *h^-^ rad57::kanMX4 (omns)^a^* | Table 2 |
| JG17751 | *h^+^ rad57::kanMX4 dbl2::natMX4 (omns)^a^* | Figure 6, Table 2 |
| JG17752 | *h^-^ rad57::kanMX4 dbl2::natMX4 (omns)^a^* | Figure 6, Table 2 |
| JG17823 | *h^+^ rad52::kanMX4 (omns)^a^* | Table 2 |
| JG17824 | *h^-^ smt0 rad52::kanMX4 (omns)^a^* | Table 2 |
| JG17747 | *h^-^ smt0 rad52::kanMX4 dbl2::natMX4 (omns)^a^* | Figure 6, Table 2 |
| JG17748 | *h^+^ rad52::kanMX4 dbl2::natMX4 (omns)^a^* | Figure 6, Table 2 |
| JG17544 | *h^90^ fbh1::kanMX4 (omns)^a^* | Figure 5, 6, Table 2 |
| JG17545 | *h^90^ fbh1::kanMX4 dbl2::natMX4 (omns)^a^* | Table 2 |
| JG17746 | *h^90^ sfr1::natMX4 (omns)^a^* | Table 2 |
| JG17811 | *h^90^ dbl2::hphMX4 sfr1::natMX4 (omns)^a^* | Figure 6, Table 2 |
| JG17513 | *h^90^ dmc1::kanMX4 (omns)^a^* | Table 2 |
| JG17511 | *h^90^ dmc1::kanMX4 dbl2::natMX4 (omns)^a^* | Table 2 |
| JG17716 | *h^90^ leu1-32 uraD18 ade6-216 rdh54::bleMX6* | Table 2 |
| JG17718 | *h^90^ leu1-32 uraD18 ade6-216 rdh54::bleMX6 dbl2::natMX4* | Table 2 |
| JG17775 | *h^90^ leu1-32 fbh1::kanMX4 pREP41-YFP-fbh1::LEU2^+^ (omns)^a^* | Figure 7 |
| JG17777 | *h^90^ leu1-32 fbh1::kanMX4 dbl2::natMX4 pREP41-YFP-fbh1::LEU2^+^ (omns)^a^* | Figure 7 |
| JG17831 | *h^90^ leu1-32*  sfr1*::natMX4* *pREP41-YFP-fbh1::LEU2^+^ (omns)^a^* | Figure 7 |
| JG17832 | *h^90^ leu1-32 sfr1::cloNAT dbl2::hph pREP41-YFP-fbh1::LEU2^+^ (omns)^a^* | Figure 7 |
| JG17833 | *h^+^ leu1-32 rad55::kanMX4 pREP41-YFP-fbh1::LEU2^+^ (omns)^a^* | Figure 7 |
| JG17834 | *h^+^ leu1-32 rad55::kanMX4 dbl2::natMX4 pREP41-YFP-fbh1::LEU2^+^ (omns)^a^* | Figure 7 |
| JG17835 | *h^+^ leu1-32 rad57::kanMX4 pREP41-YFP-fbh1::LEU2^+^ (omns)^a^* | Figure 7 |
| JG17836 | *h^+^ leu1-32 rad57::kanMX4 dbl2::natMX4 pREP41-YFP-fbh1::LEU2^+^ (omns)^a^* | Figure 7 |
| JG17837 | *h^+^ leu1-32 rad51::hphMX4 pREP41-YFP-fbh1::LEU2^+^ (omns)^a^* | Figure 7 |
| JG17838 | *h^+^ leu1-32 rad51::hphMX4 dbl2::natMX4 pREP41-YFP-fbh1::LEU2^+^ (omns)^a^* | Figure 7 |
| JG17839 | *h^+^ leu1-32 rad52::kanMX4 pREP41-YFP-fbh1::LEU2^+^ (omns)^a^* | Figure 7 |
| JG17840 | *h^+^ leu1-32 rad52::kanMX4 dbl2::natMX4 pREP41-YFP-fbh1::LEU2^+^ (omns)^a^* | Figure 7 |
| JG17841 | *h^+^ leu1-32 rad54::kanMX4 pREP41-YFP-fbh1::LEU2^+^ (omns)^a^* | Figure 7 |
| JG17842 | *h^+^ leu1-32 rad54::kanMX4 dbl2::natMX4 pREP41-YFP-fbh1::LEU2^+^ (omns)^a^* | Figure 7 |
| JG17843 | *h^-^ leu1-32 ura4-D18 ade6-M210 pREP41-YFP-fbh1::LEU2^+^* | Figure 7 |
| JG17844 | *h^-^ leu1-32 ura4-D18 ade6-M210 dbl2::natMX4 pREP41-YFP-fbh1::LEU2^+^* | Figure 7 |
| JG17961 | *h^90^ fbh1::kanMX4 dbl2-YFP::LEU2^+^ (omns)^a^* | Figure 7 |
| JG17962 | *h^90^* h90 *leu1-32* ura4D18 ade6-216 *dbl2-YFP:LEU2^+^* | Figure 7 |
| JG18021 | *h^90^ leu1-32 ura4D18 ade6-216 pREP41-YFP-fbh1::LEU2^+^* | Figure 8 |
| JG18022 | *h^90^ leu1-32 ura4D18 ade6-216 dbl2::natMX4 pREP41-YFP-fbh1::LEU2^+^* | Figure 8 |
| JG17271 | *h^90^ ade6-M216 leu1-32 lys1-131 ura4-D18 sod2[::kanr-ura4^+^-lacOp] his7^+^::lacI-GFP dbl2::natMX4* | Figure S1 |
| JG17130 | *h^90^ ade6-M216 leu1-32 lys1-131 ura4-D18 cen2(D107)[::kanr-ura4^+^-lacOp] his7^+^::lacI-GFP dbl2::natMX4* | Figure S1 |
| JG12619 | *h^90^ ade6-M216 leu1-32 lys1-131 ura4-D18 sod2[::kanr-ura4^+^-lacOp] his7^+^::lacI-GFP* | Figure S1 |
| JG12618 | *h^90^ ade6-M216 leu1-32 lys1-131 ura4-D18 cen2(D107)[::kanr-ura4^+^-lacOp] his7^+^::lacI-GFP* | Figure S1 |
| JG13990 | *h^90^ rec8-GFP::KanR* | Figure S2 |
| JG17236 | *h^90^ rec8-GFP::KanR dbl2::natMX4* | Figure S2 |
| JG11355 | *h^90^ leu1-32 ura4D18 ade6-216* | Figure S3 |
| JG17146 | *h^90^ leu1-32 ura4D18 ade6-216 dbl2::natMX4* | Figure S3 |
| JG17148 | *h^-^ leu1-32 ura4-D18 ade6-M210 dbl2::natMX4* | Figure S6 |
| JG17465 | *h^+^ ade6-M216 ura4-D18 leu1-32 eme1::kanMX4* | Figure S6 |
| JG11318 | *h^-^ leu1-32 ura4-D18 ade6-M210* | Figure S6 |
| JG17460 | *h^90^ ade6-M216 leu1-32 ura4-D18 rad22-mCherry::hphMX4 atb2-GFP::kanMX6* | Figure S5 |
| JG17510 | *h^90^ ade6-M216 leu1-32 ura4-D18 rad22-mCherry::hphMX4 atb2-GFP::kanMX6* *dbl2::natMX4* | Figure S5 |
| JG17961 | *h^90^ leu1-32 fbh1::kanMX4 pREP41-YFP-dbl2::LEU2^+^ (omns)^a^* | Figure 7, Table S6 |
| JG17962 | *h^90^ leu1-32 ura4D18 ade6-216 pREP41-YFP-dbl2::LEU2^+^* | Figure 7, Table S6 |
| GP13 | *h^-^ ade6-52* | Table 1 |
| GP1293 | *h^+^ ade6-M26 arg1-14* | Table 1 |
| GP8696 | *h^-^ ade6-52 dbl2::natMX4* | Table 1 |
| GP8698 | *h^+^ ade6-M26 arg1-14 dbl2::natMX4* | Table 1 |
| GP8450 | *h^+^ ade6-3049 dbl2::natMX4 pat1-114 rad50S* | Figure 4A |
| GP6656 | *h^-^/h^-^ bub1-234/+ ade6-3049/ade6-3049* +*/vtc4-1104* *pat1-114/pat1-114* *mbs1-24/+ +/mbs1-25 lys3-37/+ +/ura1-61 his4-239/+ +/lys4-95* | Figures 4 and S4 |
| GP8664 | *h^-^/h^-^ bub1-234/+ ade6-3049/ade6-3049* +*/vtc4-1104* *pat1-114/pat1-114* *mbs1-24/+ +/mbs1-25 lys3-37/+ +/ura1-61 his4-239/+ +/lys4-95 dbl2::natMX4/dbl2::natMX4* | Figures 4 and S4 |
| GP8836 | *h^-^/h^-^ bub1-234/+ ade6-3049/ade6-3049* +*/vtc4-1104* *pat1-114/pat1-114* *mbs1-24/+ +/mbs1-25 lys3-37/+ +/ura1-61 his4-239/+ +/lys4-95 dbl2::natMX4/dbl2::natMX4 rec12-169::kanMX6/rec12-169::kanMX6* | Figures 4 and S4 |
| GP3718 | *h^+^ ade6-3049 rad50S pat1-114 end1-458* | Figure 4 |
| JG17993 | *h^-^ smt-0 rad51::hphMX4 (omns)^a^* | Figure S10 |
| PJ69-4a | *MAT****a*** *leu2-3,112 ura3-52 trp1-901 his3-200 gal4Δ gal80Δ GAL-ADE2 lys2::GAL1-HIS3 met2::GAL7-LacZ* | Figure S9 |

^a^ omns: other auxotrophic markers not scored.
