# Supplementary material for: Dbl2 Regulates Rad51 and DNA Joint Molecule Metabolism to Ensure Proper Meiotic Chromosome Segregation
Source: PLoS Genet. 2016 Jun 15;12(6):e1006102. doi: 10.1371/journal.pgen.1006102 (PMC4909299; doi:10.1371/journal.pgen.1006102)
Supplement: S2 Table — (DOCX) [file pgen.1006102.s012.docx]

**Table S2. Holliday junctions are formed and repaired similarly in wild-type and *dbl2Δ* mutant, but Rec12-dependent joint molecules persist in *dbl2Δ* late meiosis – analysis at the *mbs1* DSB hotspot.**

|  | 0 hr | 2 hr | 3 hr | 4 hr | 5 hr | 6 hr | 7 hr | 8 hr |
| --- | --- | --- | --- | --- | --- | --- | --- | --- |
| *dbl2^+^* ex 1 | 1.9 | 9.6 | 0.3 | 2.6 | 0.4 | 0.07 |  | 0.02 |
| *dbl2^+^* ex 2 | 0.7 | 7 | 0.7 | 2.1 | 0.3 | 0.03 | 0.1 | 0.04 |
| *dbl2^+^* ex 3 | 0.6 | 6.9 | 0.9 | 2.5 | 0.9 | 0.2 | 0.1 |  |
| *dbl2^+^* ex 4 | 1.5 |  |  | 2.7 | 0.2 | 0.07 | 0.06 |  |
| *dbl2^+^* ex 5 | 0.9 |  |  | 3.3 | 0.1 | 0.08 | 0.01 |  |
| *dbl2^+^* ex 6 |  |  |  |  |  | 0.07 |  |  |
|  |  |  |  |  |  |  |  |  |
| mean | 1.1 | 7.8 | 0.6 | 2.6 | 0.4 | 0.09 | 0.07 | 0.03 |
| SD | 0.5 | 1.5 | 0.3 | 0.4 | 0.3 | 0.06 | 0.04 | 0.01 |
| SEM | 0.2 | 0.9 | 0.2 | 0.2 | 0.1 | 0.02 | 0.02 | 0.01 |
|  |  |  |  |  |  |  |  |  |
| *dbl2Δ *ex 1 | 0.4 | 7.5 | 1 | 3 | 0.4 | 0.2 | 0.2 | 0.3 |
| *dbl2Δ* ex 2 | 0.5 | 4.8 | 1.1 | 1.6 | 0.2 | 0.3 | 0.4 | 0.2 |
| *dbl2Δ* ex 3 | 0.5 | 5.6 | 1.2 | 2 | 0.7 | 0.3 | 0.3 |  |
| *dbl2Δ* ex 4 | 0.9 |  |  | 2.6 | 0.6 | 0.3 | 0.2 |  |
| *dbl2Δ* ex 5 | 1 |  |  | 3.4 | 0.4 | 0.3 |  |  |
| *dbl2Δ* ex 6 |  |  |  |  |  | 0.3 |  |  |
|  |  |  |  |  |  |  |  |  |
| mean | 0.7 | 6 | 1.1 | 2.5 | 0.5 | 0.3 | 0.3 | 0.2 |
| SD | 0.3 | 1.4 | 0.1 | 0.7 | 0.2 | 0.04 | 0.09 | 0.05 |
| SEM | 0.1 | 0.8 | 0.06 | 0.3 | 0.08 | 0.02 | 0.05 |  |
|  |  |  |  |  |  |  |  |  |
| *rec12Δ dbl2Δ* ex 1 | 0.6 | 8.4 | 0.8 | 0.1 | 0.1 | 0.08 | 0.01 | 0.08 |
| *rec12Δ dbl2Δ* ex 1 | 1.8 | 7.1 | 1 | 0.2 | 0.05 | 0.01 | 0.06 | 0.1 |
|  |  |  |  |  |  |  |  |  |
| Mean | 1.2 | 7.7 | 0.9 | 0.15 | 0.07 | 0.04 | 0.03 | 0.09 |
| Range/2 | 0.6 | 0.6 | 0.1 | 0.05 | 0.02 | 0.03 | 0.02 | 0.01 |

Data are the percent of total DNA detected as joint molecules at the indicated times after meiotic induction of strains GP6656 (*dbl2^+^*), GP8664 (*dbl2Δ*), and GP8836 (*dbl2Δ rec12Δ*). Data are from Figures 4B and S4B and in two to six additional experiments (ex).
