# Supplementary material for: Dbl2 Regulates Rad51 and DNA Joint Molecule Metabolism to Ensure Proper Meiotic Chromosome Segregation
Source: PLoS Genet. 2016 Jun 15;12(6):e1006102. doi: 10.1371/journal.pgen.1006102 (PMC4909299; doi:10.1371/journal.pgen.1006102)
Supplement: S3 Table — (DOCX) [file pgen.1006102.s013.docx]

|  | 0 hr | 2 hr | 3 hr | 4 hr | 5 hr | 6 hr | 7 hr | 8 hr |
| --- | --- | --- | --- | --- | --- | --- | --- | --- |
| *dbl2^+^* ex1 | 0.9 | 16.5 | 0.6 | 3.3 | 0.5 | 0.07 | 0.07 | 0.01 |
| *dbl2^+^* ex2 | 2 | 25 | 1.1 | 3.5 | 0.5 | 0.01 | 0.07 | 0.01 |
| *dbl2^+^* ex3 | 0.8 | 24 | 1.1 | 6 | 0.2 | 0.1 |  |  |
|  |  |  |  |  |  |  |  |  |
|  |  |  |  |  |  |  |  |  |
| Mean | 1.2 | 21.8 | 0.9 | 4.3 | 0.4 | 0.06 | 0.07 | 0.01 |
| SD | 0.7 | 4.6 | 0.3 | 1.5 | 0.2 | 0.04 | 0 | 0 |
| SEM | 0.4 | 2.7 | 0.2 | 0.9 | 0.1 | 0.03 |  |  |
|  |  |  |  |  |  |  |  |  |
| *dbl2Δ* ex1 | 0.7 | 18 | 1.8 | 3.6 | 0.6 | 0.3 | 0.2 | 0.2 |
| *dbl2Δ* ex2 | 1.2 | 18 | 1.1 | 4 | 0.7 | 0.3 | 0.4 | 0.3 |
| *dbl2Δ* ex3 | 1.4 | 19.8 | 1.1 | 5.3 | 0.6 | 0.3 |  |  |
|  |  |  |  |  |  |  |  |  |
|  |  |  |  |  |  |  |  |  |
| mean | 1.1 | 18.6 | 1.3 | 4.3 | 0.6 | 0.3 | 0.3 | 0.2 |
| SD | 0.4 | 1.0 | 0.4 | 0.9 | 0.06 | 0 | 0.1 | 0.05 |
| SEM | 0.2 | 0.6 | 0.2 | 0.5 | 0.03 | 0 |  |  |
|  |  |  |  |  |  |  |  |  |
| *rec12Δ dbl2Δ* ex1 | 1 | 30 | 0.6 | 0.1 | 0.1 | 0.1 | 0.06 | 0.03 |
| *rec12Δ dbl2Δ* ex2 | 1.5 | 31 | 0.5 | 0.2 | 0.03 | 0.1 | 0.1 | 0.09 |
|  |  |  |  |  |  |  |  |  |
| Mean | 1.2 | 30.5 | 0.5 | 0.1 | 0.06 | 0.1 | 0.08 | 0.06 |
| Range/2 | 0.2 | 0.5 | 0.05 | 0.05 | 0.3 | 0 | 0.02 | 0.03 |

**Table S3. Holliday junctions are formed and repaired similarly in wild-type and *dbl2Δ* mutant, but Rec12-dependent joint molecules persist in *dbl2Δ* late meiosis – analysis at the *ade6-3049* DSB hotspot.**

Data are the percent of total DNA detected as joint molecules at the indicated times after meiotic induction of strains GP6656 (*dbl2^+^*), GP8664 (*dbl2Δ*), and GP8836 (*dbl2Δ rec12Δ*). Data are from Figures S4A and B and in two or three additional experiments (ex).
