# Supplementary material for: Dbl2 Regulates Rad51 and DNA Joint Molecule Metabolism to Ensure Proper Meiotic Chromosome Segregation
Source: PLoS Genet. 2016 Jun 15;12(6):e1006102. doi: 10.1371/journal.pgen.1006102 (PMC4909299; doi:10.1371/journal.pgen.1006102)
Supplement: S4 Table — (DOCX) [file pgen.1006102.s014.docx]

**Table S4. Dbl2 is required for efficient targeting of Fbh1 to DNA lesions induced by CPT.**

|  | Cells with Fbh1-YFP foci (%) **(40 µM CPT)** | | |
| --- | --- | --- | --- |
|  | Experiment 1 | Experiment 2 | Experiment 3 |
| *wt* | 26 | 28 | 30 |
| *dbl2Δ* | 8 | 6 | 6 |

*S. pombe* strains expressing Fbh1-YFP from plasmid pMW651 and carrying *fbh1Δ* (JG17775) or *fbh1Δ dbl2Δ* (JG17777) mutations growing in EMM2 medium without leucine were treated with 40 µM CPT for 4 hr and fixed; DNA was visualized with DAPI. Fbh1-YFP foci were scored in three sets of 200 G2 cells.
