# Supplementary material for: Dbl2 Regulates Rad51 and DNA Joint Molecule Metabolism to Ensure Proper Meiotic Chromosome Segregation
Source: PLoS Genet. 2016 Jun 15;12(6):e1006102. doi: 10.1371/journal.pgen.1006102 (PMC4909299; doi:10.1371/journal.pgen.1006102)
Supplement: S5 Table — (DOCX) [file pgen.1006102.s015.docx]

**Table S5. Dbl2 is required for efficient targeting of Fbh1 to DNA lesions induced by deletion of genes involved in homologous recombination.**

|  | Cells with Fbh1-YFP foci (%)  **(20 µM CPT)** | | | Cells with Fbh1-YFP foci (%)  **(no CPT added)** | | |
| --- | --- | --- | --- | --- | --- | --- |
|  | Experiment 1 | Exp. 2 | Exp. 3 | Exp. 1 | Exp. 2 | Exp. 3 |
| *wt* | 10 | 8 | 7 | 1 | 1 | 1 |
| *dbl2Δ* | 2 | 2 | 5 | 1 | 1 | 1 |
| *rad51Δ* | 29 | 43 | 35 | 10 | 13 | 20 |
| *rad51Δ dbl2Δ* | 10 | 10 | 8 | 7 | 1 | 1 |
| *rad52Δ* | 40 | 40 | 38 | 20 | 38 | 22 |
| *rad52Δ dbl2Δ* | 6 | 7 | 8 | 4 | 4 | 8 |
| *rad55Δ* | 33 | 30 | 29 | 20 | 21 | 10 |
| *rad55Δ dbl2Δ* | 5 | 6 | 4 | 6 | 9 | 5 |
| *rad57Δ* | 30 | 36 | 33 | 26 | 27 | 21 |
| *rad57Δ dbl2Δ* | 6 | 4 | 5 | 4 | 1 | 9 |
| *sfr1Δ* | 20 | 7 | 14 | 12 | 12 | 10 |
| *sfr1Δ dbl2Δ* | 3 | 5 | 4 | 3 | 5 | 1 |
| *rad54Δ* | 15 | 18 | 14 | 17 | 13 | 15 |
| *rad54Δ dbl2Δ* | 12 | 15 | 8 | 3 | 2 | 3 |

*S. pombe* strains expressing Fbh1-YFP from plasmid pMW651 growing in EMM2 medium without leucine were treated with or without 20 µM CPT for 4 hr and fixed, and examined by fluorescence microscopy; DNA was visualized with DAPI. Fbh1-YFP foci were scored in three sets of 200 G2 cells. The strains used were wild-type (JG17843), *dbl2Δ* (JG17844), *rad51Δ* (17837), *rad51Δ dbl2Δ* (JG17838), *rad52Δ* (JG17839), *rad52Δ dbl2Δ* (JG17840), *rad55Δ* (JG17833), *rad55Δ dbl2Δ* (JG17834), *rad57Δ* (JG17835), *rad57Δ dbl2Δ* (JG17836), *sfr1Δ* (JG17831), *sfr1Δ dbl2Δ* (JG17832), *rad54Δ* (JG17841), *rad54Δ dbl2Δ* (JG17842).
