# Supplementary material for: Dbl2 Regulates Rad51 and DNA Joint Molecule Metabolism to Ensure Proper Meiotic Chromosome Segregation
Source: PLoS Genet. 2016 Jun 15;12(6):e1006102. doi: 10.1371/journal.pgen.1006102 (PMC4909299; doi:10.1371/journal.pgen.1006102)
Supplement: S6 Table — (DOCX) [file pgen.1006102.s016.docx]

**Table S6. Dbl2-YFP forms foci independently of Fbh1 in cells with DNA lesions induced by CPT or MMS.**

|  | Cells with Dbl2-YFP foci (%) | | |
| --- | --- | --- | --- |
|  | Experiment 1 | Experiment 2 | Experiment 3 |
| wt | 39 | 49 | 45 |
| *fbh1Δ* | 59 | 63 | 63 |
| *wt* (+ 25 µM CPT) | 63 | 59 | 58 |
| *fbh1Δ* (+ 25 µM CPT) | 66 | 75 | 62 |
| *wt* (+ 0.005% MMS) | 89 | 86 | 72 |
| *fbh1Δ* (+ 0.005% MMS) | 86 | 83 | 78 |

*S. pombe* wild-type strain (JG17962) and *fbh1Δ* (JG17961) mutant expressing Dbl2-YFP were grown in EMM2 medium without leucine and treated with either 25 µM CPT or 0.005% MMS for 4 hr and fixed, and examined by fluorescence microscopy; DNA was visualized with DAPI. Dbl2-YFP foci were scored in three sets of 200 G2 cells. Dbl2-YFP was expressed from *nmt1* promoter from the plasmid SpYFH25C07 [5] integrated into the genome.
