# Supplementary material for: Dbl2 Regulates Rad51 and DNA Joint Molecule Metabolism to Ensure Proper Meiotic Chromosome Segregation
Source: PLoS Genet. 2016 Jun 15;12(6):e1006102. doi: 10.1371/journal.pgen.1006102 (PMC4909299; doi:10.1371/journal.pgen.1006102)
Supplement: S1 References — (DOCX) [file pgen.1006102.s017.docx]

**Supporting Information**

**Dbl2 regulates Rad51 and DNA joint molecule metabolism to ensure proper meiotic chromosome segregation**

Silvia Polakova^1,2,#,*^, Lucia Molnarova^3,#^, Randy W. Hyppa^4^, Zsigmond Benko^2^, Ivana Misova^1^, Alexander Schleiffer^5^, Gerald R. Smith^4,*^ and Juraj Gregan^2,3,*^

^1^ Department of Membrane Biochemistry, Institute of Animal Biochemistry and Genetics, Slovak Academy of Sciences, Ivanka pri Dunaji, Slovakia

^2^ Department of Chromosome Biology, MFPL, University of Vienna, Vienna, Austria

^3^ Department of Genetics, Faculty of Natural Sciences, Comenius University, Bratislava, Slovakia

^4^ Division of Basic Sciences, Fred Hutchinson Cancer Research Center, Seattle, Washington, United States of America

^5^ IMP/IMBA Bioinformatics core facility, Research Institute of Molecular Pathology (IMP), Vienna Biocenter, Vienna, Austria

^#^ Equal contributions

^*^ Corresponding authors, E-mails: silvia.polakova@univie.ac.at, gsmith@fhcrc.org, gregan@fns.uniba.sk

**Supplementary References**

1. Hyppa RW, Fowler KR, Cipak L, Gregan J, Smith GR (2014) DNA intermediates of meiotic recombination in synchronous *S. pombe* at optimal temperature. Nucleic Acids Res 42: 359-369.

2. Katoh K, Toh H (2008) Recent developments in the MAFFT multiple sequence alignment program. Brief Bioinform 9: 286-298.

3. Waterhouse AM, Procter JB, Martin DM, Clamp M, Barton GJ (2009) Jalview Version 2--a multiple sequence alignment editor and analysis workbench. Bioinformatics 25: 1189-1191.

4. Palecek J, Vidot S, Feng M, Doherty AJ, Lehmann AR (2006) The Smc5-Smc6 DNA repair complex. bridging of the Smc5-Smc6 heads by the KLEISIN, Nse4, and non-Kleisin subunits. J Biol Chem 281: 36952-36959.
